# Rhamnellosides A and B, ω-Phenylpentaene Fatty Acid Amide Diglycosides from the Fruits of *Rhamnella franguloides*

**DOI:** 10.3390/molecules23040752

**Published:** 2018-03-24

**Authors:** Kyo Bin Kang, Ming Gao, Geum Jin Kim, Hyukjae Choi, Sang Hyun Sung

**Affiliations:** 1 College of Pharmacy and Research Institute of Pharmaceutical Sciences, Seoul National University, Seoul 08826, Korea; kbinkang@gmail.com; 2 College of Pharmacy, Yeungnam University, Gyeongsan 38541, Korea; gaoming104@hotmail.com (M.G.); kimgeumjin@naver.com (G.J.K.)

**Keywords:** *Rhamnella franguloides*, Rhamnaceae, ω-phenylpentaene fatty acid amide, dereplication

## Abstract

Two new ω-phenylpentaene fatty acid amide diglycosides, rhamnellosides A (**1**) and B (**2**), were isolated from the fruits of *Rhamnella franguloides* (Rhamnaceae). These compounds were prioritized using LC-MS/MS molecular networking dereplication based on our previous discovery of 2-acetoxy-ω-phenylpentaene fatty acid triglycosides berchemiosides A−C from a phylogenetically related species, *Berchemia berchemiifolia*. The structures of the isolated compounds were elucidated by spectroscopic analyses in combination with chemical derivatization. The pentaene groups of **1** and **2** were found to have (6*E*, 8*E*, 10*Z*, 12*Z*, 14*E*)-geometry, which is the same as that found in berchemioside A.

## 1. Introduction

**Figure 1:**
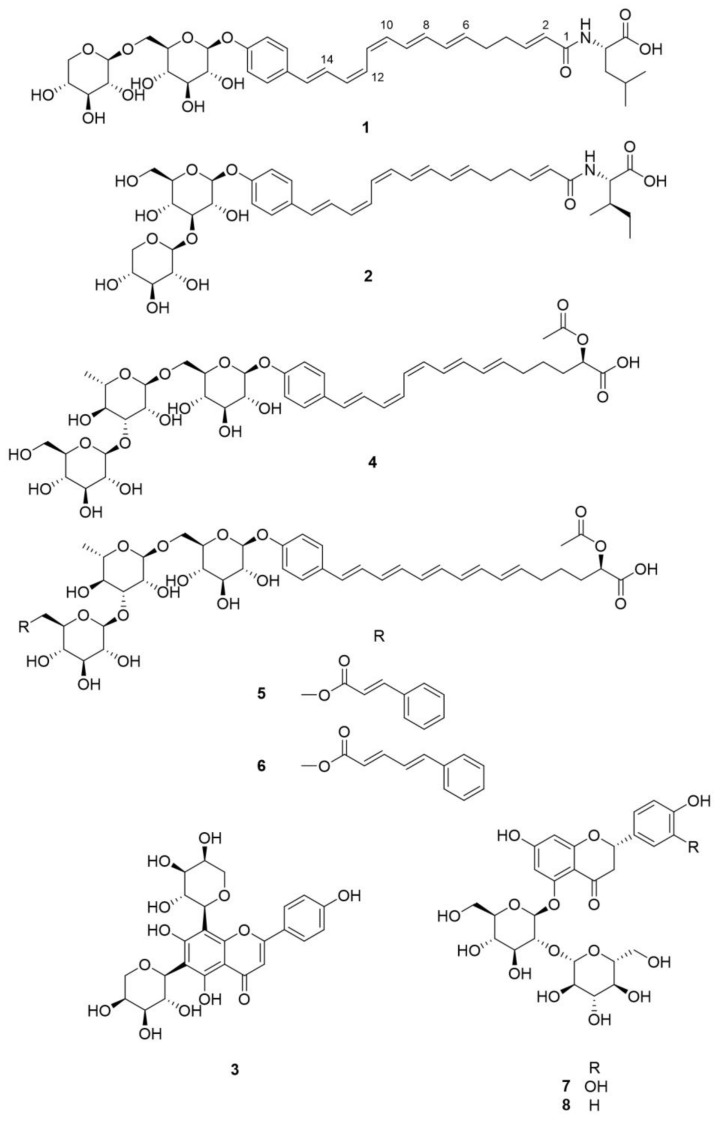
Chemical structures of compounds **1**–**3** isolated from *R. franguloides* and **4**–**8** previously isolated from *B. berchemiifolia*.

The genus *Rhamnella* (family Rhamnaceae) comprises approximately 10 species of evergreen shrubs and small trees, which are found in central China, Japan, Korea, and the Himalayas [1]. One of those species, *R. gilgitica*, has been used for the treatment of rheumatism, swelling, and pain in traditional Chinese medicines [2], but the other species are not known to have any commercial uses. Little is known about the phytochemicals in the *Rhamnella* species; only a few flavonoids and fatty acids have been reported from *R. gilgitica* [3,4] and *R. inaequilatera* [5]. In this study, we report the targeted isolation and structural elucidation of two ω-phenylpentaene fatty acid amide diglycosides, rhamnelloside A (**1**) and B (**2**), along with a previously known flavonoid di-*C*-glycoside (**3**), from the fruits of *R. franguloides*, the only *Rhamnella* species found in Korea (Figure 1). Based on our previous discovery of 2-acetoxy-ω-phenylpentaene fatty acid triglycosides berchemiosides A–C (**4**–**6**) from the fruits of *Berchemia berchemiifolia* (Rhamnaceae) [6], we hypothesized that phytochemicals with similar structures would be present in species that are genetically similar to *B. berchemiifolia*. *R. franguloides* was selected for the follow-up study because plastid DNA sequence-based analysis revealed that *Rhamnella* is phylogenetically close to *Berchemia* [7]. Fruits of *R. franguloides* look similar to those of *B. berchemiifolia*; they are 0.8- to 1.5-cm long drupes and are yellow when immature. However, unlike those of *B. berchemiifolia*, the fruits of *R. franguloides* turn black when they are fully mature. For dereplication and prioritization, MS/MS molecular networking, an emerging tool for the exploration of MS/MS data from natural products [8,9], was applied to prioritize the ω-phenylpentaene fatty acid derivatives in *R. franguloides*. This method establishes an MS/MS spectral similarity network, which allows an efficient investigation of structurally similar metabolites [10].

## 2. Results and Discussion

**Figure 2:**
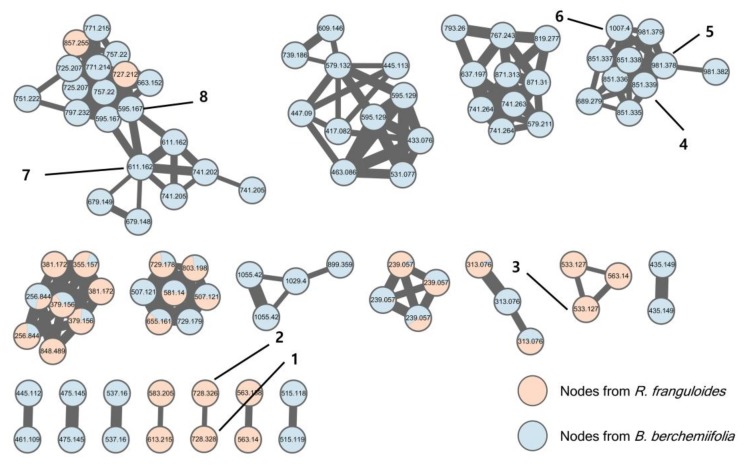
MS/MS molecular network of *R. franguloides* and *B. berchemiifolia* fruit extracts. Red nodes represent ions from *R. franguloides*; blue nodes represent ions from *B. berchemiifolia*. Only clusters containing at least two nodes are shown.

Contrary to our expectation, none of the spectral nodes from the *R. franguloides* extract were clustered with compounds **4**–**6** in the molecular network, which was established based on UHPLC-Q/TOF-MS data from the *B. berchemiifolia* and *R. franguloides* extracts (Figure 2). Only two spectral nodes from *R. franguloides* could be putatively annotated by a clustering with nodes from *B. berchemiifolia*; nodes in a cluster with compounds **7** and **8** were suggested to be flavanone 5-*O*-diglycosides based on their spectral similarity to **7** and **8**. This kind of dissimilarity between the two species was unexpected, so we decided to focus on the dissimilarity instead. Spectral clusters that formed nodes with compounds only from *R. franguloides* were carefully investigated. A cluster containing three nodes of precursor ions at *m/z* 533.127 and 563.140 was initially examined; tandem MS spectra of those nodes exhibited characteristic neutral losses of *m/z* 60, 90, 120, and 150 Da, suggesting that these nodes correspond to flavonoid di-*C*-glycosides [11]. This was confirmed by the targeted isolation of compound **3**, which was identified as apigenin 6,8-di-*C*-α-l-arabinopyranoside by spectral comparison with a reference sample [12]. Afterward, two spectral nodes, both with precursor ions of *m/z* 728.3287 ([M − H]^−^, calcd for C_38_H_50_NO_13_, 728.3288), were prioritized because their even *m/z* values suggested they were alkaloids. These spectral nodes exhibited two chromatographic peaks with similar retention times to those of **4**–**6** (Figure S1, Supplementary Materials). The characteristic broad UV absorption spectra of these peaks suggested that they are derivatives of conjugated phenylpolyene fatty acids. The targeted isolation of these prioritized peaks yielded previously unknown compounds **1** and **2**.

**Figure 3:**
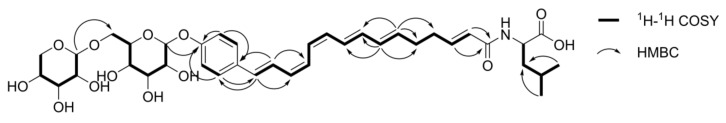
Key ^1^H-^1^H COSY and HMBC correlations of compound **1**.

Compound **1** was isolated as a yellow, amorphous solid. As described above, the HRESIMS data suggested its molecular formula was C_38_H_51_NO_13_. The MS/MS spectrum of **1** exhibited fragment ions at *m/z* 434.2336 ([M − C_11_H_18_O_9_ − H]^−^, calcd for C_27_H_32_NO_4_, 434.2337) and 130.0865 ([C_6_H_12_NO_2_]^−^, calcd 130.0874). The former was thought to correspond to the loss of a hexose unit and a pentose unit, and the latter suggested that **1** is a fatty acid amide derivative with a leucine or isoleucine moiety. Acid hydrolysis followed by thin layer chromatography (TLC) and HPLC analysis after arylthiocarbamoyl-thiazolidine derivatization [13] confirmed the assignments of a d-glucosyl (Glc) unit and a d-xylosyl (Xyl) unit. Using C_3_ Marfey’s method [14] on the hydrolysate confirmed the presence of an l-leucine unit. The ^1^H NMR spectrum of **1** confirmed that **1** is a ω-phenylpentaene fatty acid derivative based on the presence of an aromatic A_2_B_2_ spin system (δ_H_ 7.41 (2H, d, *J* = 8.7 Hz, H-17 and H-21) and 7.02 (2H, d, *J* = 8.7 Hz, H-18 and H-20)) and 10 conjugated polyene protons at δ_H_ 5.75–6.85 (Table 1). However, the C-1–5 spin systems were different from those of the corresponding carbons in **4**; two olefinic protons at δ_H_ 6.58 (1H, m, H-3) and 6.01 (1H, d, *J* = 15.3 Hz, H-2) exhibited HMBC correlations with the amide carbonyl carbon at δ_C_ 164.5, which suggested a *trans*-α-unsaturation in **1** (Figure 3). Two methylenes at δ_H_ 2.24 (4H, br s, H-4, H-5) were also observed, and those resonances were attributed to H-4 and H-5 based on ^1^H-^1^H COSY and HMBC experiments. Coupling constants of the anomeric protons of the two sugars (δ_H_ 4.80 (1H, d, *J* = 7.6 Hz, Glc H-1) and 4.18 (1H, d, *J* = 7.5 Hz, Xyl H-1)) suggested that the d-glucose and d-xylose units were in β-configurations. The HMBC correlation between the anomeric Xyl H-1 and Glc C-6 (δ_C_ 82.0) protons suggested a 1→6 interglycosidic linkage, and the HMBC correlation between Glc H-1 and C-19 (δ_C_ 157.0) confirmed the ω-glycosylation at the phenylpentaene fatty acid amide moiety. For compounds **4**–**6**, the geometries of the conjugated double bonds were established from the two-dimensional (2D) *J*-resolved NMR spectrum [6]. Interestingly, compound **1** was suggested to have (6*E*, 8*E*, 10*Z*, 12*Z*, 14*E*)-geometry, which is identical to that of **4** (Figure S8, Supplementary Materials). Consequently, the structure of compound **1**, rhamnelloside A, was defined as 15-(4-*O*-β-d-xylopyranosyl-(1→6)-β-d-glucopyranosylphenyl)-pentadeca-2*E*,6*E*,8*E*,10*Z*,12*Z*,14*E*-hexaenoic acid l-leucinamide.

Compound **2** had a molecular formula of C_38_H_51_NO_13_, which is the same as that of **1**. The MS/MS spectrum of **2** was also similar to that of **1**; it showed a cosine similarity of 0.6378 in the molecular network and exhibited fragment ions at *m/z* 434.2345 [M − C_11_H_18_O_9_ − H]^−^ and 130.0852 [C_6_H_12_NO_2_]^−^. Some regions of the one-dimensional (1D) and 2D NMR spectra of **2** were not clear due to the small amount of **2** available (0.2 mg), but compound **2** could be confidently assigned to also be an ω-phenylpentaene fatty acid amide derivative. In its ^1^H NMR spectrum, two methyl group signals at δ_H_ 0.84 (3H, d, *J* = 6.2 Hz, Ile H-5) and 0.83 (3H, t, *J* = 7.0 Hz, Ile H-6) suggested that **2** had an isoleucine moiety instead of the leucine moiety found in **1** (Table 1). Acid hydrolysis and use of C_3_ Marfey’s method revealed the absolute configuration of the amino acid residue in **2** as being l-*allo*-isoleucine, a nonproteinogenic amino acid that naturally occurs in plants, fungi, and human plasma [15]. The acid hydrolysis of **2** also revealed the presence of a d-glucosyl unit and a d-xylosyl unit. However, the resonances of Glc H-6 (δ_H_ 3.36 and 3.28) indicated that compound **2** did not have a 1→6 interglycosidic linkage. Otherwise, the downfield-shifted Glc H-3 (δ_H_ 3.46) suggested the presence of a 1→3 interglycosidic linkage, and this assignment was confirmed by the HMBC correlation between Xyl H-1 (δ_H_ 4.28 (1H, d, *J* = 7.6 Hz)) and Glc C-3 (δ_C_ 78.8). Compound **2** was also identified to have (6*E*, 8*E*, 10*Z*, 12*Z*, 14*E*)-geometry based on the *J*-resolved NMR spectrum (Figure S16, Supplementary Materials). Thus, rhamnelloside B (**2**) was defined as 15-(4-*O*-β-d-xylopyranosyl-(1→3)-β-d-glucopyranosylphenyl)-pentadeca-2*E*,6*E*,8*E*,10*Z*,12*Z*,14*E*-hexaenoic acid l-*allo*-isoleucinamide.

Isolated compound **1** and previously isolated compounds **4**, **5**, and **6** were evaluated for their antimicrobial activities against *Bacillus subtilis*, *Escherichia coli* DH5α, *Pseudomonas aeruginosa*, and *Staphylococcus aureus* and showed no activities (minimum inhibitory concentration > 250 μM; compound **2** could not be tested because of the scarce amount).

## 3. Materials and Methods 

### 3.1. General Experimental Procedures

Optical rotations were measured on a JASCO P-2000 polarimeter using a 1-cm cell. UV and electronic circular dichroism (ECD) spectra were recorded on a Chirascan CD spectrometer (Applied Photophysics, Surrey, UK). 1D and 2D NMR spectra were obtained with Bruker AVANCE III HD 850 spectrometers (Bruker, Billerica, MA, USA) at the National Center for Interuniversity Research Facilities at Seoul National University (NCIRF). UHPLC-Q/TOF-MS analyses were performed on a Waters Acquity UPLC system (Waters Co., Milford, MA, USA) coupled with a Waters Xevo G2 QTOF mass spectrometer (Waters MS Technologies, Manchester, UK) that was equipped with an electrospray interface (ESI). The absolute configurations of the amino acids in compounds **1** and **2** were determined using an Agilent 6120 quadruple MSD consisting of a 1260 Infinity pump, a 1260 Infinity autosampler, a 1260 Infinity DAD (Agilent Technologies, Santa Clara, CA, USA), and an Agilent Zorbax SB-C_3_ column (150 × 4.6 mm, 5 μm) at 50 °C. Semi-preparative HPLC separations were performed with a system consisting of a Gilson 321 Pump and a UV/Vis-151 detector (Gilson Inc., Middleton, WI, USA). Extra-pure grade solvents for extraction, fractionation, and isolation were purchased from Dae Jung Pure Chemical Engineering Co. Ltd., Siheung, Korea. Deuterated DMSO for NMR analyses was purchased from Merck (Darmstadt, Germany). 

### 3.2. Plant Material

*R. franguloides* was cultivated in the Medicinal Plant Garden, College of Pharmacy, Seoul National University, Koyang, Korea (GPS N37°42′42.9′′, E126°49′10.6′′), and the ripe fruits from one individual plant were collected in September 2015. The sample was authenticated by Mr. S. I. Han (The Medicinal Plant Garden, College of Pharmacy, Seoul National University), and a voucher specimen (SUPH-1509-05) was deposited in the Herbarium of the Medicinal Plant Garden.

### 3.3. LC-MS/MS Molecular Networking

Separations were conducted on a Waters Acquity UPLC BEH C_18_ (100 mm × 2.1 mm, 1.7 μm) column. The flow rate of the mobile phase was 0.3 mL/min, and the column temperature was maintained at 30 °C. The mobile phase consisted of H_2_O (A) and MeCN (B) with a linear gradient of 10–90% B (0–20 min). Analyses of the samples (1.0 μL injected into the partial loop with needle overfill mode) were performed using an optimized data-dependent acquisition (DDA) mode consisting of a full MS survey scan in the 100–1500 Da range (scan time: 100 ms), followed by an MS/MS scan for the three most intense ions. The collision energy was applied at a gradient from 20 to 80 V. The molecular network was created using the Data Analysis workflow 2.0 on the Global Natural Products Social Molecular Networking (GNPS) platform (http://gnps.ucsd.edu) [9]; the reliability was enhanced by data preprocessing using MZmine 2 software [16]. Raw LC-MS files were converted into mzXML using ProteoWizard 3.0.9935 [17] and then imported into MZmine 2.29. The mass detection was performed with the noise level at 1000 (for MS) and 40 (for MS/MS). The chromatogram was built with ions showing a minimum time span of 0.01 min, a minimum height of 2500, and an *m/z* tolerance of 0.001 (or 5.0 ppm). The chromatographic deconvolution was achieved by a baseline cut-off algorithm, with the following parameters: minimum peak height of 1500, peak duration range of 0.02–0.15 min, and baseline level of 500. Chromatograms were deisotoped using an algorithm for grouping isotopic peaks with an *m/z* tolerance of 0.002 (or 5.0 ppm) and a t_R_ tolerance of 0.1 min. The preprocessed chromatograms were exported to GNPS for molecular networking. MS/MS spectra were window filtered by choosing only the top six peaks in the ±50 Da window throughout the spectrum. A network was then created where edges were filtered to have a cosine score above 0.60 and more than three matched peaks. Further edges between two nodes were kept in the network if and only if each of the nodes appeared in each other’s respective top 10 most similar nodes. The library spectra were filtered in the same manner as the input data. The molecular network was visualized using Cytoscape 3.5.1 [18]. The MS/MS data are deposited in the MassIVE Public GNPS data set (http://gnps.ucsd.edu, MSV000081660). 

### 3.4. Extraction and Isolation

Frozen fruits of *R. franguloides* (27.5 g including seeds) were extracted with MeOH (2 × 100 mL, for 3 h each) with ultrasonication at room temperature, and then the extract was concentrated in vacuo. The crude extract (582.9 mg) was suspended in 100 mL of H_2_O and partitioned successively (2 × 100 mL for each solvent) into CHCl_3_ (43.0 mg), EtOAc (15.1 mg), and BuOH (67.8 mg) using a separation funnel. The EtOAc fraction was subjected to semi-preparative reversed-phase HPLC on a YMC Triart C_18_ column (10 × 250 mm, 5 μm, YMC Co. Ltd., Kyoto, Japan) eluting with 45% aqueous MeCN to yield compounds **1** (1.0 mg) and **2** (0.2 mg). The BuOH fraction was purified by semi-preparative reversed-phase HPLC on a YMC Triart C_18_ column (10 × 250 mm, 5 μm, YMC Co. Ltd., Kyoto, Japan) eluting with 20% aqueous MeCN to yield compound **3** (1.5 mg).

*Rhamnelloside A (**1**):* Yellow, amorphous solid; ${\left[\alpha \right]}_{D}^{20}$ + 34.5 (*c* 0.10, MeOH); UV (MeOH) *λ*_max_ (log *ε*) 391 (3.1), 370 (2.2), 351 (3.2), 336 (3.0) nm; ECD (MeOH) *λ*_max_ (Δ) 366 (−0.6), 334 (0.1), 309 (−0.1), 282 (0.1), 229 (−1.0), 212 (0.0) nm; ^1^H and ^13^C NMR, see Table 1; HRESIMS *m/z* 728.3287 [M − H]^−^ (calcd for C_38_H_50_NO_13_, 728.3288).

*Rhamnelloside B (**2**):* Yellow, amorphous solid; ${\left[\alpha \right]}_{D}^{20}$ − 42.6 (*c* 0.10, MeOH); UV (MeOH) *λ*_max_ (log *ε*) 391 (3.3), 369 (3.3), 351 (3.2), 337 (3.0) nm; ECD (MeOH) *λ*_max_ (Δ) 375 (−0.3), 354 (−0.1), 327 (−0.3), 278 (0.0), 229 (−1.0), 211 (−0.3) nm; ^1^H and ^13^C NMR, see Table 1; HRESIMS *m/z* 728.3298 [M − H]^−^ (calcd for C_38_H_50_NO_13_, 728.3288).

### 3.5. Acid Hydrolysis and Determination of the Resulting Sugars

Approximately 0.5 mg of **1** and 0.2 mg of **2** were hydrolyzed with 1 N HCl (200 µL) at 80 °C for 2 h. The hydrolysates were extracted with EtOAc (2 × 200 µL) to remove the aglycones. The aqueous fractions were concentrated and compared to authentic d-glucose (R_f_ 0.65) and d-xylose (R_f_ 0.78) standards (Sigma-Aldrich, St. Louis, MO, USA) on silica gel TLC plates with BuOH-acetone-pyridine-H_2_O (2:2:1:1) visualized with *p*-anisaldehyde. The absolute configurations of the sugars in the hydrolysates were determined by HPLC analysis after arylthiocarbamoyl-thiazolidine derivatization. The aqueous fractions of the hydrolysates were dissolved in pyridine (100 µL) containing l-cysteine methyl ester hydrochloride (0.5 mg), and the mixtures were heated at 60 °C for 1 h. *o*-Tolyl isothiocyanate (100 µL, Tokyo Chemical Industry Co., Ltd., Tokyo, Japan) was then added, and the mixtures were heated at 60 °C for an additional 1 h. The reaction mixtures were directly analyzed on a Dionex Ultimate 3000 HPLC system (Thermo Fisher Scientific Inc., Waltham, MA, USA). The separation was performed on a Waters XBridge C_18_ column (250 × 4.6 mm, 5 μm) eluting with a mixture of H_2_O (A) and MeCN (B) with a linear gradient of 20–40% B (0–30 min). The column temperature was maintained at 30.0 °C. Authentic d-glucose, l-glucose, d-xylose, and l-xylose were also prepared and analyzed using the same procedure. The retention times of the hydrolysates and sugar derivatives were as follows: d-glucose derivative (t_R_ 14.59 min), l-glucose derivative (t_R_ 13.87 min), d-xylose derivative (t_R_ 15.81 min), and l-xylose derivative (t_R_ 15.24 min).

### 3.6. Determination of the Absolute Configurations of the Amino Acids in Compounds 1 and 2

The hydrolysates (50 μg) of compound **1** were added to 1 M NaHCO_3_ (20 µL) with 1% l-FDAA (N^α^-(5-fluoro-2,4-dinitrophenyl)-l-alaninamide, Tokyo Chemical Industry Co., Ltd.) in acetone (40 µL). The reaction vials were incubated and stirred for 1 h at 40 °C. The reactions were then quenched with 1 N HCl (20 μL) and prepared for LC-MS analysis. The samples were analyzed by LC-MS on an Agilent Zorbax SB-C_3_ column (150 × 4.6 mm, 5 μm) at 50 °C with a gradient of 25% B to 65% B (95% MeOH+5% MeCN (1% formic acid)) (0–75 min) in A (H_2_O). The retention times of the hydrolysates and authentic leucine derivatives were as follows: hydrolysate derivative-l-FDAA (t_R_ 40.746 min), l-Leu-l-FDAA (t_R_ 40.306 min), and d-Leu-l-FDAA (t_R_ 53.233 min). The leucine unit in compound **1** was confirmed to be l-Leu.

The hydrolysates of compound **2** were characterized using the same method as that used for compound **1**. The LC-MS conditions were as follows: 25% B to 45% B (95% MeOH + 5% MeCN (1% formic acid)) (0–75 min) and 100% B at 77 min, where A is H_2_O. The retention times of the hydrolysates and authentic isoleucine derivatives were as follows: hydrolysate derivative-l-FDAA (t_R_ 59.468 min), l-allo-Ile-l-FDAA (t_R_ 59.383 min), l-Ile-l-FDAA (t_R_ 61.362 min), d-allo-Ile-l-FDAA (t_R_ 78.819 min), and d-Ile-l-FDAA (t_R_ 79.172 min). The isoleucine unit in compound **2** was determined to be l-allo-Ile.

### 3.7. Antimicrobial Assay

The broth microdilution method was used to evaluate the antimicrobial activities of the isolated compounds [19]. Four bacterial strains (*Bacillus subtilis*, *Escherichia coli* DH5α, *Pseudomonas aeruginosa*, and *Staphylococcus aureus*) were inoculated in Muller-Hinton (MH) agar plates and suspended in MH broth with 0.5 McFarland turbidity equivalents. The prepared microorganism suspensions were diluted with MH broth. The concentrations of the positive control (gentamicin) and tested compounds were 20 mM in DMSO. The stock solution of the compounds diluted with MH broth medium (50 μL) were mixed with 50 μL of microorganism suspensions diluted to concentrations between 250 and 0.5 μM using serial two-fold dilutions in 96-well plates. The experiments were performed in triplicate. The 96-well plates were incubated for 14 h at 27 °C for *B. subtilis* and at 37 °C for *E. coli* DH5α, *P. aeruginosa*, and *S. aureus*. The minimum inhibitory concentrations were determined as the lowest concentrations that visually inhibited the growth of the microorganism.

## 4. Conclusions

In summary, the discovery of two new ω-phenylpentaene fatty acid amide diglycosides, rhamnellosides A (**1**) and B (**2**) from *R. franguloides* is described in this work. Based on our previous discovery of 2-acetoxy-ω-phenylpentaene fatty acid triglycosides (**4**–**6**) from a phylogenetically close species *B. berchemiifolia*, metabolites in the fruits of *R. franguloides* were chemically screened using an LC-MS/MS molecular networking dereplication strategy. Although compounds **1** and **2** were not directly annotated by molecular networking, they were prioritized based on LC-MS/MS spectra and isolated. The structures of **1** and **2** were identified by analyses in combination with chemical derivatization. Interestingly, compounds **1** and **2** have unprecedented (6*E*, 8*E*, 10*Z*, 12*Z*, 14*E*)-geometry in their pentaene groups, which is the same as **4** from *B. berchemiifolia*. Although these compounds did not show significant activity in the biological evaluation for antibacterial activity, these unprecedented metabolites would have biological or ecological roles, which should be revealed by further studies.

## Figures and Tables

**Table 1:** The ^1^H (850 MHz) and ^13^C (212.5 MHz) NMR spectroscopic data (δ in ppm) of compounds **1** and **2** (DMSO-*d*_6_).

Position	1		2	
	δ_H_ mult. (*J* in Hz)	δ_C_^a^	δ_H_ mult. (*J* in Hz)	δ_C_^a^
1		164.5		n.d. ^c^
2	6.01, d (15.3)	124.9	6.03, d (15.4) ^b^	125.7
3	6.58, m	141.7	6.56, m	141.3
4	2.24, br s	31.1	2.23, br s	31.6
5	2.24, br s	31.1	2.23, br s	31.6
6	5.75, dt (15.0, 7.1)	134.3	5.78, dt (15.2, 7.6) ^b^	134.6
7	6.17, dd (15.0, 9.8)	131.4	6.17, dd (15.2, 10.3) ^b^	131.4
8	6.27, dd (16.3, 9.8) ^b^	133.6	6.29, dd (15.1, 10.3) ^b^	133.5
9	6.28, dd (16.3, 8.1) ^b^	131.5	6.29, dd (15.1, 9.0) ^b^	131.7
10	6.35, dd (9.0, 8.1) ^b^	132.9	6.37, dd (9.6, 9.0) ^b^	133.3
11	6.44, dd (9.0, 9.0) ^b^	133.1	6.45, dd (9.6, 9.6) ^b^	133.4
12	6.35, dd (9.0, 9.0) ^b^	132.9	6.37, dd (9.6, 9.6) ^b^	133.3
13	6.44, dd (9.6, 9.0)	133.1	6.45, dd (10.5, 9.6) ^b^	133.4
14	6.85, dd (15.4, 9.6)	127.7	6.87, dd (15.2, 10.5) ^b^	128.3
15	6.56, d (15.4)	131.9	6.58, d (15.2) ^b^	132.2
16		131.1		131.6
17	7.41, d (8.7)	127.6	7.41, d (8.5)	127.8
18	7.02, d (8.7)	116.6	6.98, d (8.5)	116.8
19		157.0		157.3
20	7.02, d (8.7)	116.6	6.98, d (8.5)	116.8
21	7.41, d (8.7)	127.6	7.41, d (8.5)	127.8
Leu (**1**)/Ile (**2**)				
1		172.5		n.d. ^c^
2	3.98, m	52.9	4.01, m	n.d. ^c^
3	1.50, m; 1.37, m	43.0	1.59, m	25.0
4	1.58, m	25.1	1.36, m; 1.23, m	29.2
5	0.84, d (2.3)	22.8	0.84, d (6.2) ^b^	23.5
6	0.84, d (2.3)	23.7	0.83, t (7.0) ^b^	22.9
Glc				
1	4.80, d (7.6)	100.4	4.95, d (7.6)	100.2
2	3.23, m	73.7	3.29, m	69.7
3	3.27, m	76.9	3.46, m	78.8
4	3.14, m	70.2	3.14, m	70.0
5	3.53, m	76.3	3.52, m	75.9
6	3.96, d (10.9); 3.55, m	68.7	3.36, m; 3.28, m	63.5
Xyl				
1	4.18, d (7.5)	104.4	4.28, d (7.6)	103.6
2	2.99, m	73.9	2.99, m	74.0
3	3.07, m	77.0	3.11, m	76.9
4	3.26, m	70.1	3.27, m	69.9
5	3.67, m; 2.95, m	66.1	3.76, m; 3.08, m	65.9

^a 13^C NMR spectra were unavailable because of the scarce number of isolates; hence, chemical shifts were suggested by HSQC and HMBC spectra.; ^b^ *J* values were approximately identified based on *J*-resolved NMR spectra.; ^c^ Not determined in HSQC or HMBC spectra due to the scarce amount of purified **2**.

## Supplementary Materials

The following are available online, LC-MS BPI chromatograms of the *R. franguloides* and *B. berchemiifolia* fruit extracts; raw HRESIMS (MS and MS/MS), NMR, UV, and ECD data for compounds **1** and **2**.
