# Supplementary material for: Rhamnellosides A and B, ω-Phenylpentaene Fatty Acid Amide Diglycosides from the Fruits of Rhamnella franguloides
Source: Molecules. 2018 Mar 24;23(4):752. doi: 10.3390/molecules23040752 (PMC6017831; doi:10.3390/molecules23040752)
Supplement: Supplementary file 1 [file molecules-23-00752-s001.pdf]

Supplementary Data for

Rhamnellosides A and B, ω-Phenylpentaene Fatty Acid Amide Diglycosides from the Fruits of *Rhamnella franguloides*

Kyo Bin Kang,[1,†] Ming Gao,[2,†] Geum Jin Kim,[2] Hyukjae Choi,[2,*] and Sang Hyun Sung[1,*]

[1] College of Pharmacy and Research Institute of Pharmaceutical Sciences, Seoul National University, Seoul 08826, Republic of Korea

[2] College of Pharmacy, Yeungnam University, Gyeongsan 38541, Republic of Korea

[†] These authors equally contributed to this work.

*Correspondence to:

Sang Hyun Sung (shsung@snu.ac.kr)

Hyukjae Choi (h5choi@yu.ac.kr)

Table of Contents

Figure S1. UHPLC–Q/TOF–MS base peak ion (BPI) chromatograms of methanol extracts of (a) *B. berchemiifolia* and (b) *R. franguloides* fruits. Peak numbers designate the isolated compounds **1–8**.

Figure S2. The MS spectrum of **1**

Figure S3. The MS/MS spectrum of **1**

Figure S4. The ^{1}H NMR (850 MHz, DMSO-d_6) spectrum of **1**

Figure S5. The ^{1}H-^{1}H COSY (850 MHz, DMSO-d_6) spectrum of **1**

Figure S6. The HSQC (850 MHz, DMSO-d_6) spectrum of **1**

Figure S7. The HMBC (850 MHz, DMSO-d_6) spectrum of **1**

Figure S8. The *J*-resolved (850 MHz, DMSO-d_6) spectrum of **1**, expanded for the polyene region.

Figure S9. UV and CD spectra of **1**

Figure S10. The MS spectrum of **2**

Figure S11. The MS/MS spectrum of **2**

Figure S12. The ^{1}H NMR (850 MHz, DMSO-d_6) spectrum of **2**

Figure S13. The ^{1}H-^{1}H COSY (850 MHz, DMSO-d_6) spectrum of **2**

Figure S14. The HSQC (850 MHz, DMSO-d_6) spectrum of **2**

Figure S15. The HMBC (850 MHz, DMSO-d_6) spectrum of **2**

Figure S16. The *J*-resolved (850 MHz, DMSO-d_6) spectrum of **2**, expanded for the polyene region.

Figure S17. UV and CD spectra of **2**

Figure S18. TLC for determining sugar moieties in hydrolysates of **1** (a) and **2** (b). Standards of glucose, arabinose, and xylose were spotted together.

Figure S19. HPLC chromatograms for analyzing sugar derivatives in hydrolysates of **1** and **2**. (a) D-glucose standard derivative (b) (c) D-xylose standard derivative (d) L-xylose standard derivative (e) derivatized hydrolysate of compound **1** (f) derivatized hydrolysate of compound **2**.

Figure S20. HPLC-DAD/MS chromatograms in C3 Marfey's analyses on the Leu unit of the hydrolysate of **1**. (a) L-FDAA derivatized L/D mixture of Leu, (b) L-FDAA

derivatized hydrolysate of **1**.

Figure S21. HPLC-UV (340 nm) chromatograms in C3 Marfey's analyses on the Leu and Ile units of the hydrolysate of **2**. (a) L-FDAA derivatized L/D standard mixtures of Leu/Ile/*allo*-Ile, (b) L-FDAA derivatized hydrolysate of **2**, (c) co-injected L-FDAA derivatized the standard mixture and hydrolysate of **2**.

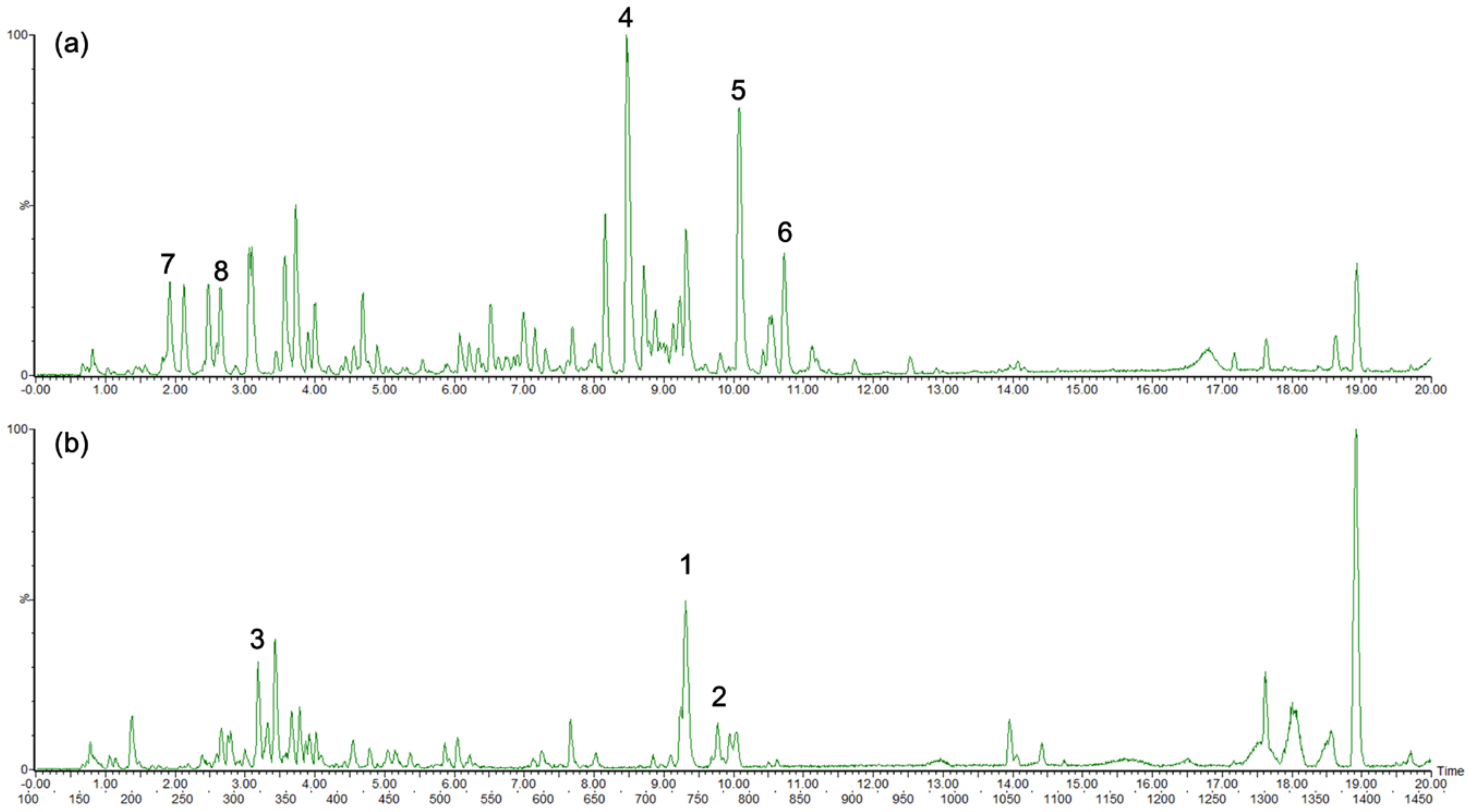

Figure S1. UHPLC–Q/TOF–MS base peak ion (BPI) chromatograms of methanol extracts of (a) *B. berchemiifolia* and (b) *R. franguloides* fruits. Peak numbers designate compounds **1–8**.

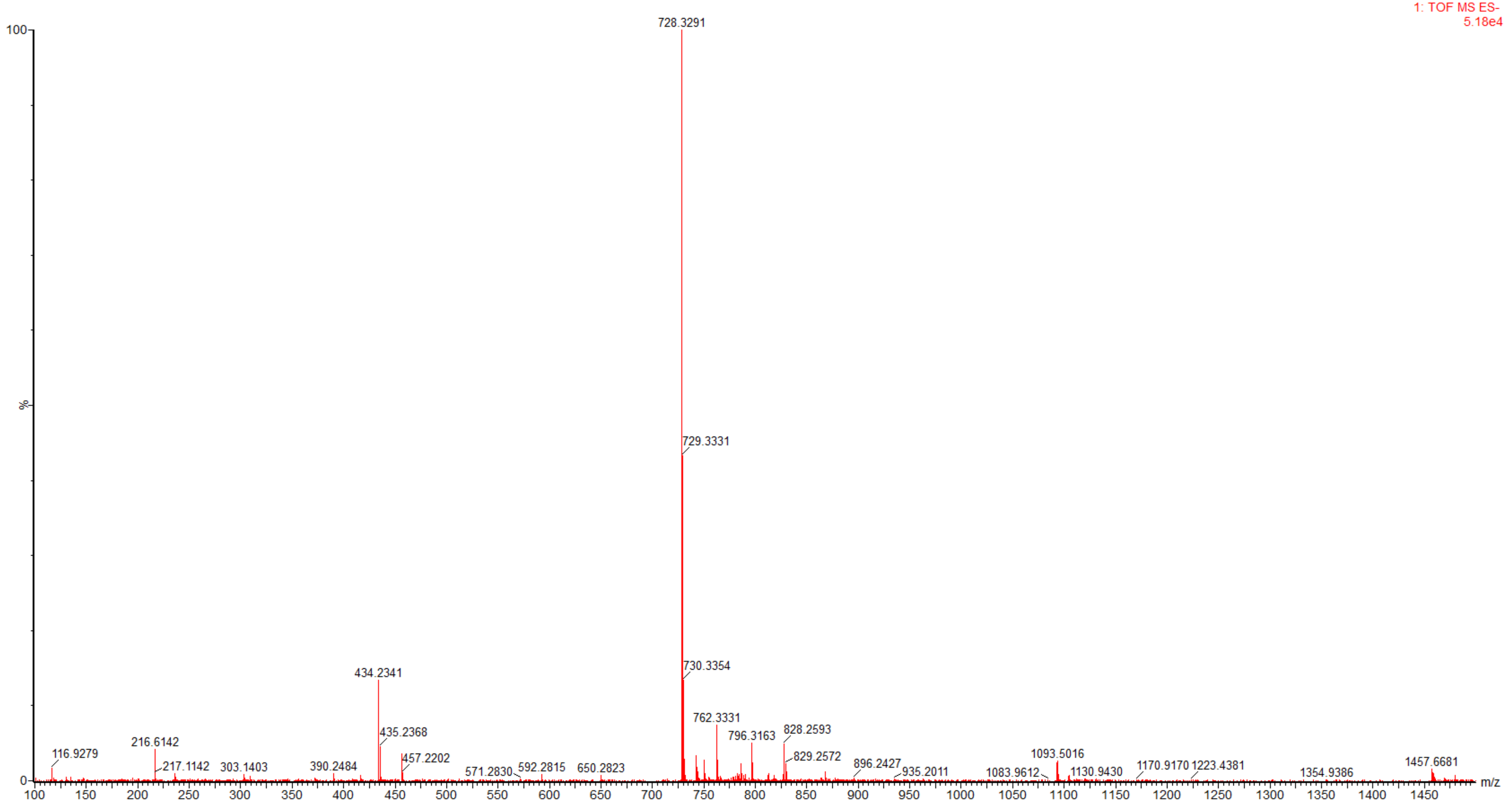

Figure S2. The MS spectrum of **1**

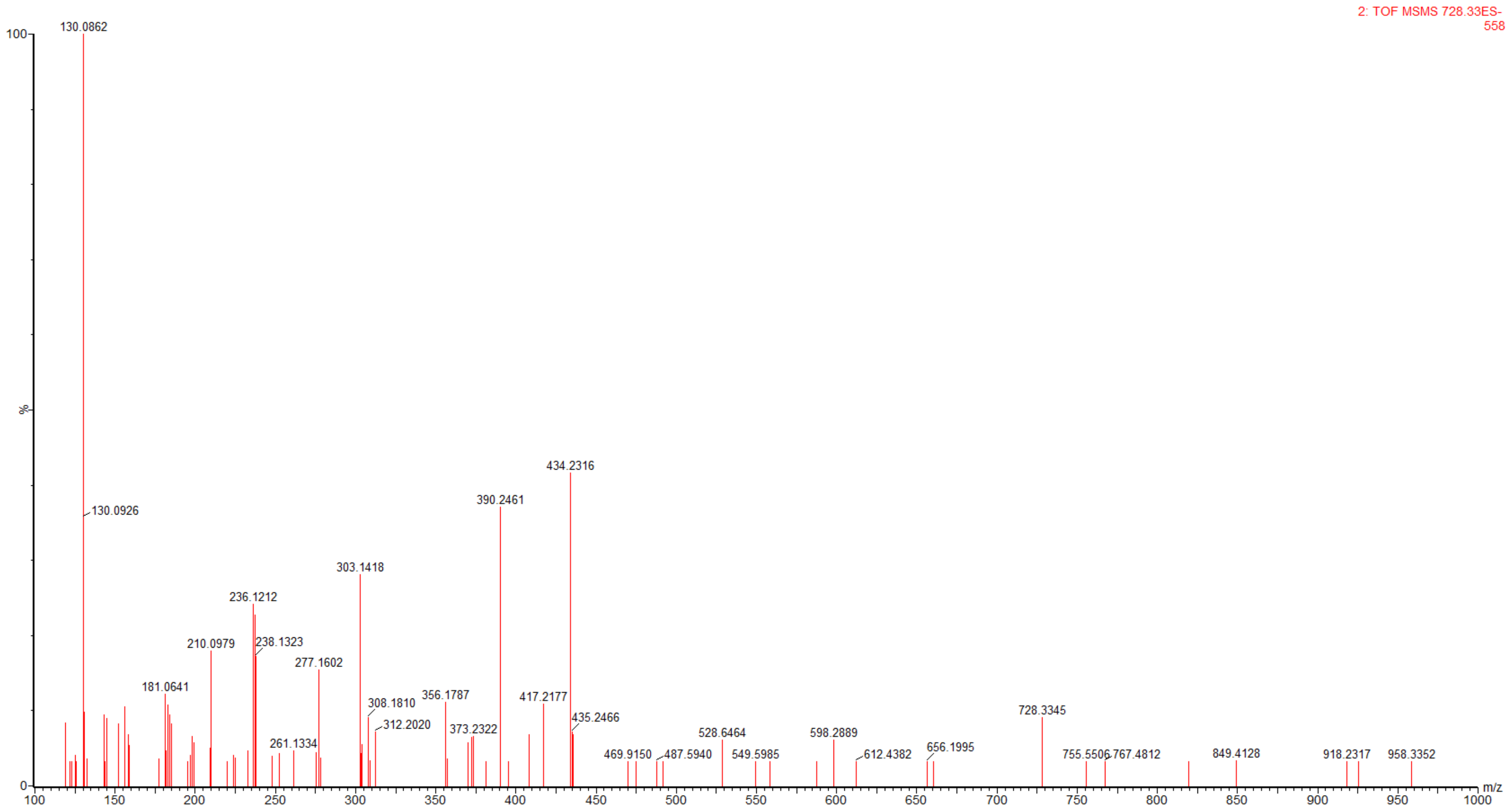

Figure S3. The MS/MS spectrum of **1**

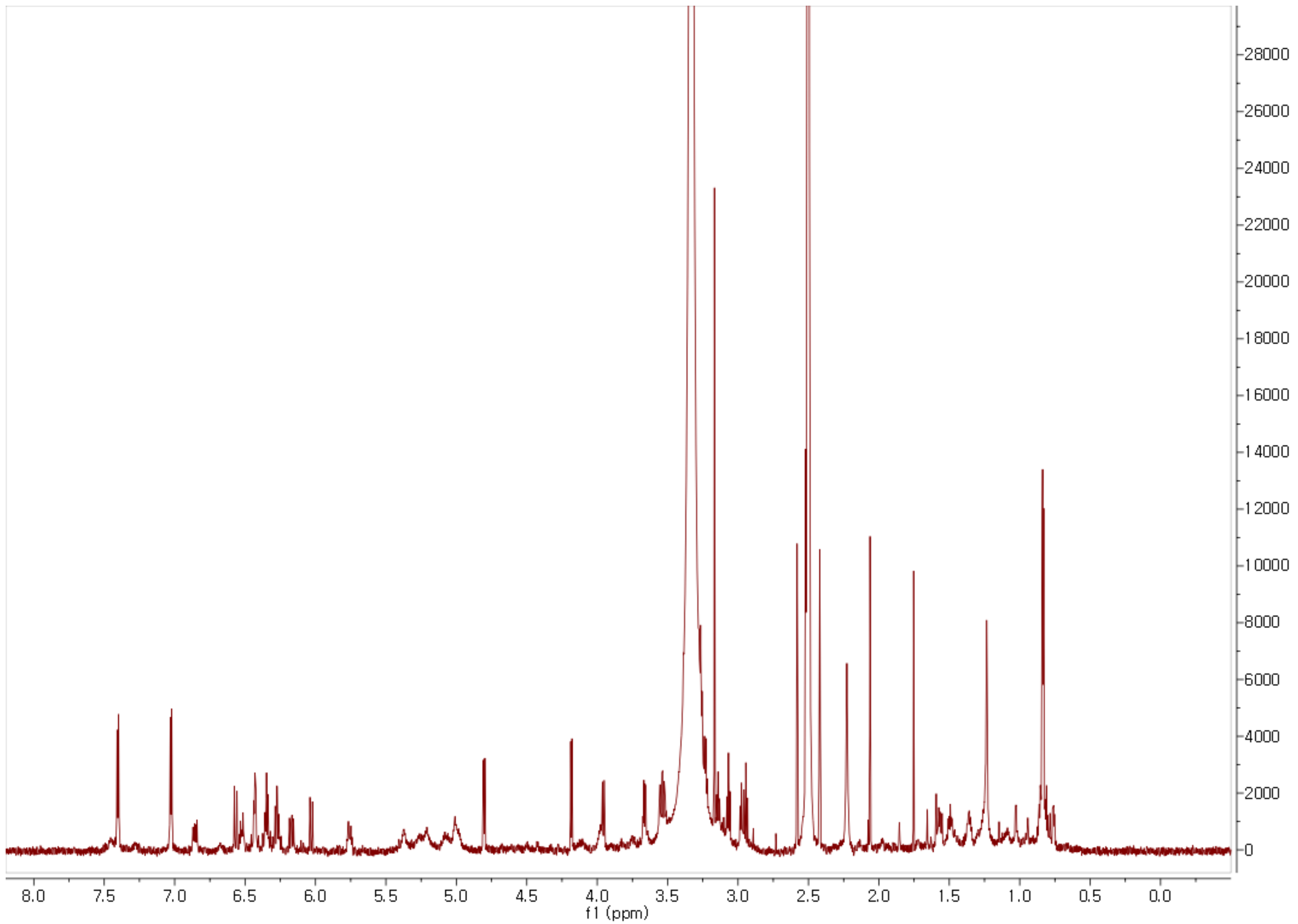

Figure S4. The ^{1}H NMR (850 MHz, DMSO-d_6) spectrum of **1**

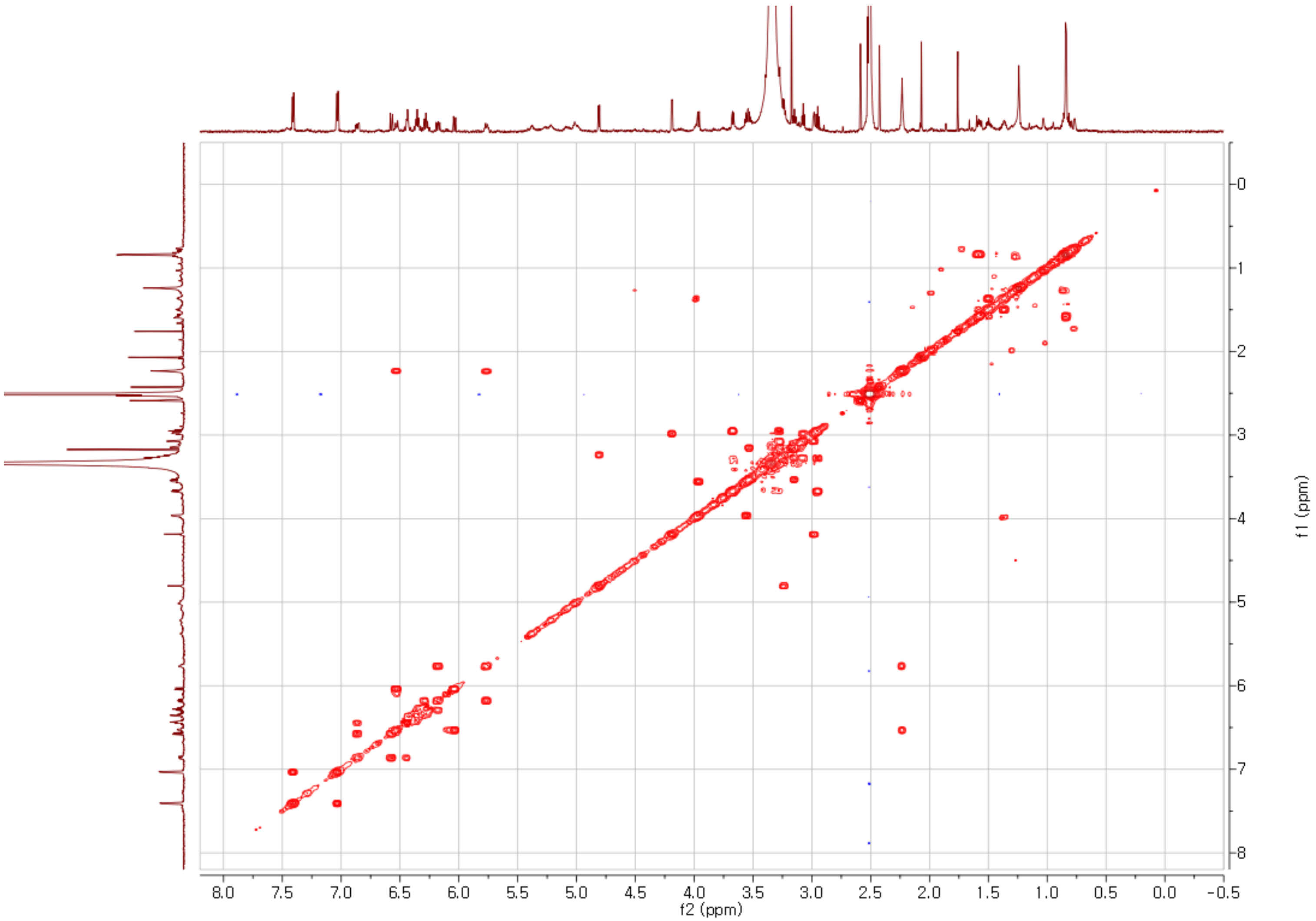

Figure S5. The ^{1}H-^{1}H COSY (850 MHz, DMSO-d_6) spectrum of **1**

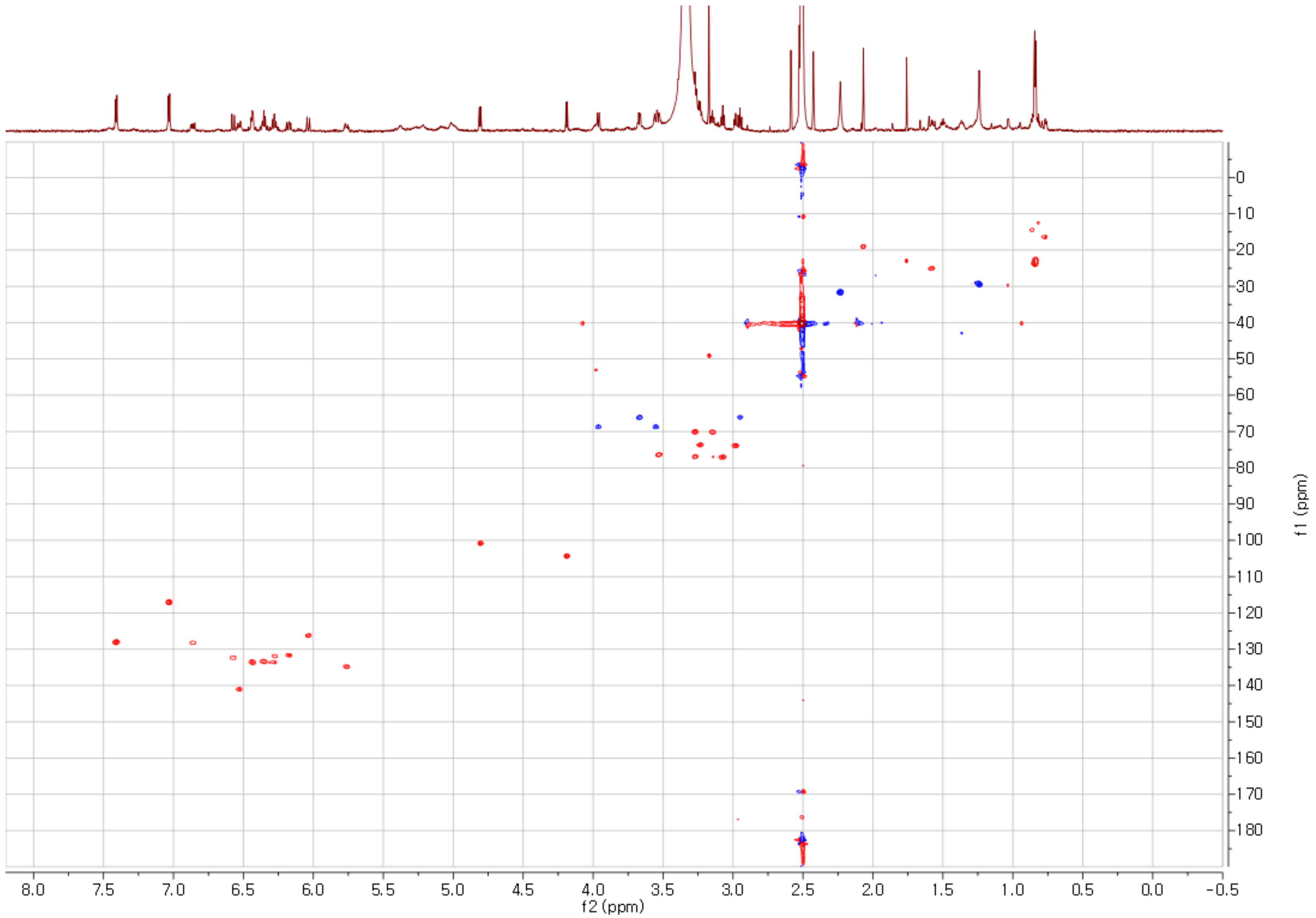

Figure S6. The HSQC (850 MHz, DMSO-d_6) spectrum of **1**

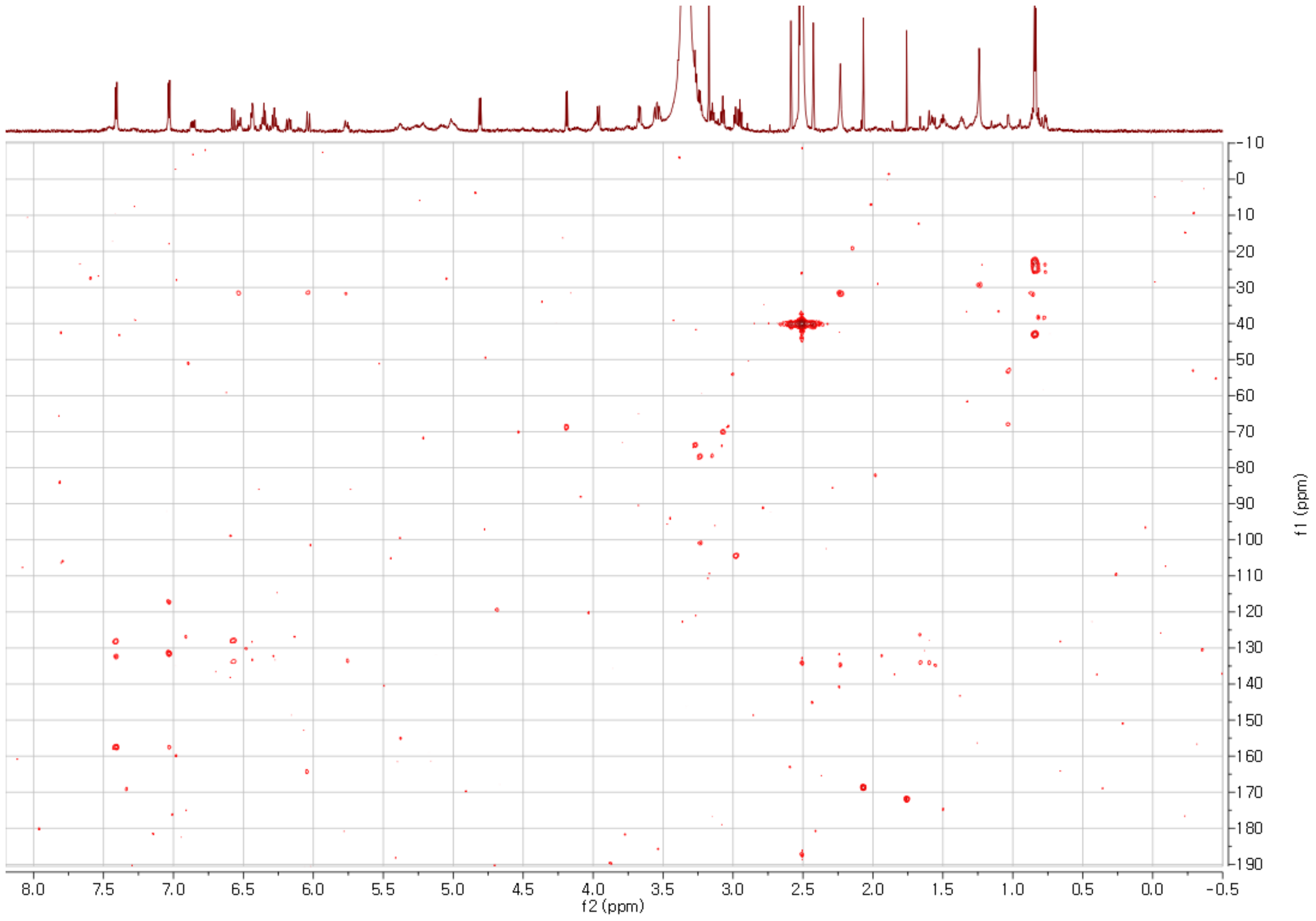

Figure S7. The HMBC (850 MHz, DMSO-d_6) spectrum of **1**

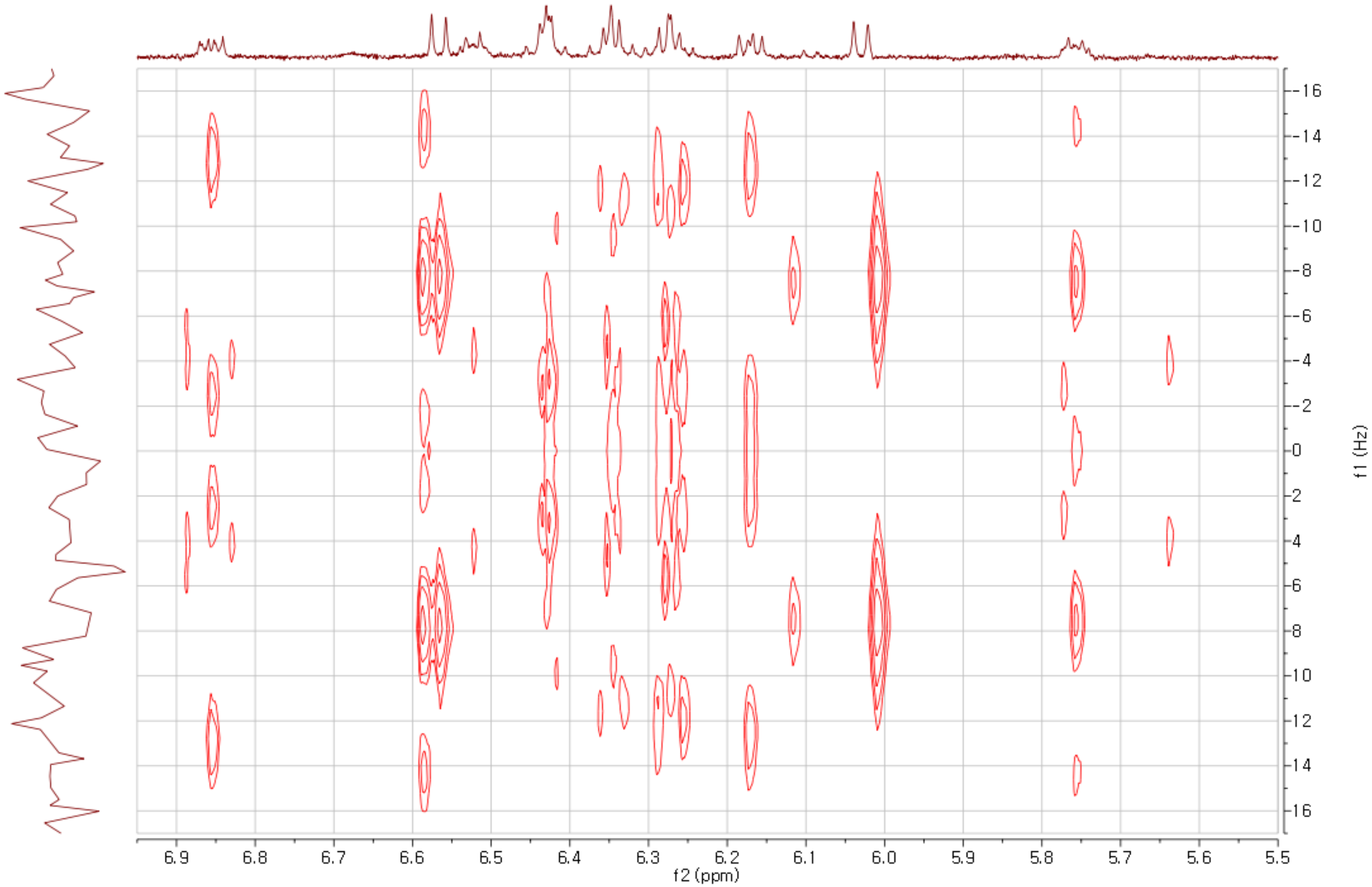

Figure S8. The *J*-resolved (850 MHz, DMSO-d_6) spectrum of **1**, expanded for the polyene region.

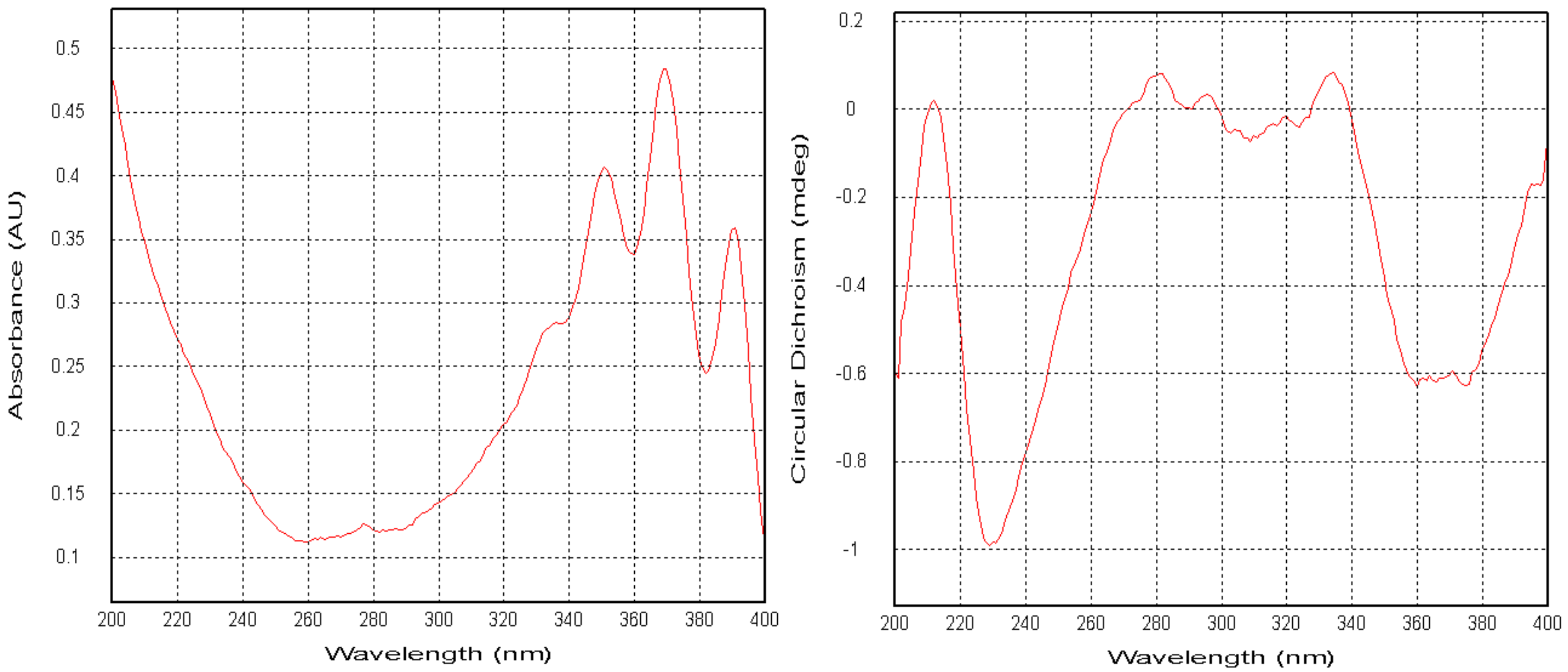

Figure S9. UV and CD spectra of **1**

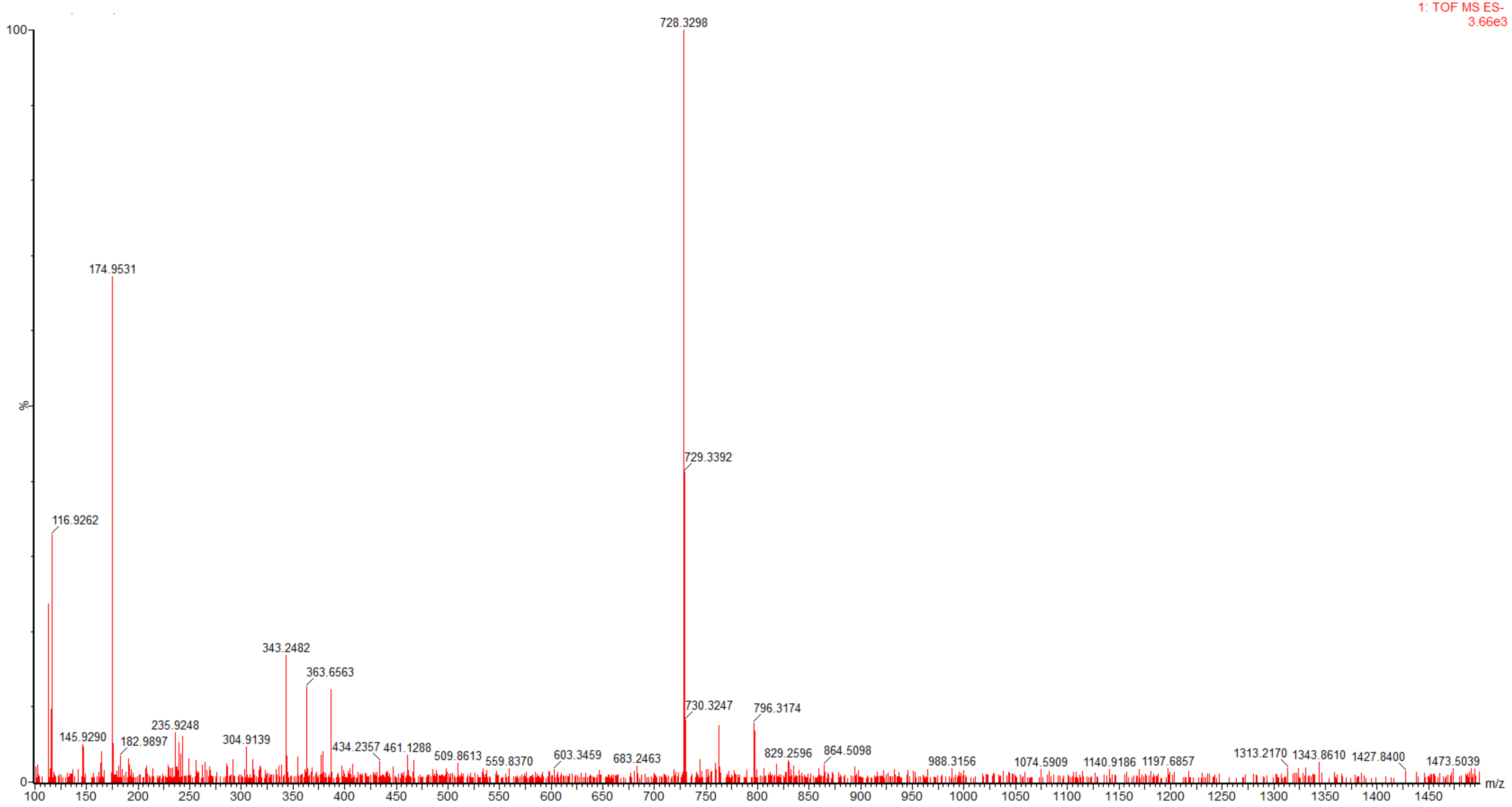

Figure S10. The MS spectrum of **2**

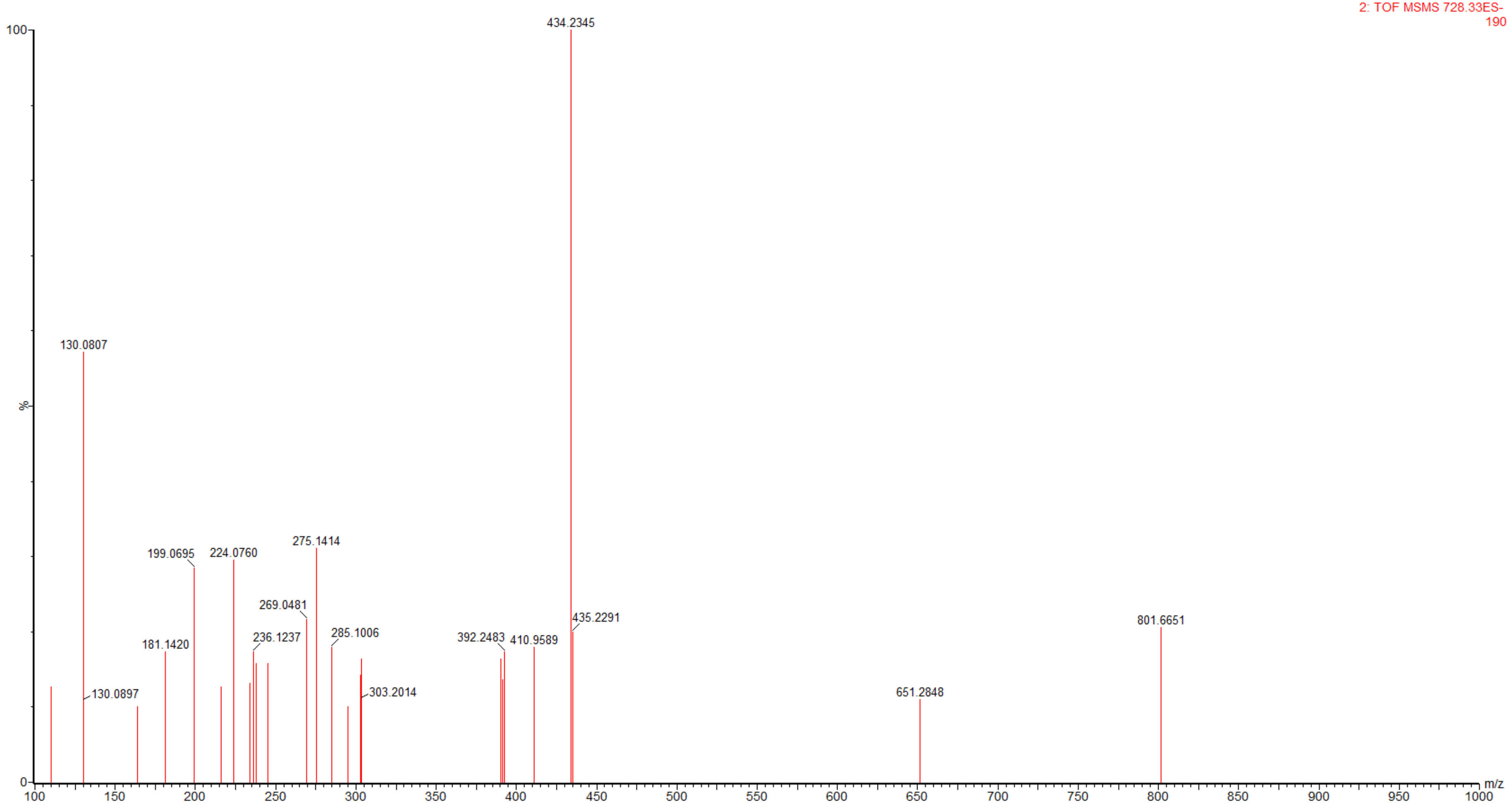

Figure S11. The MS/MS spectrum of **2**

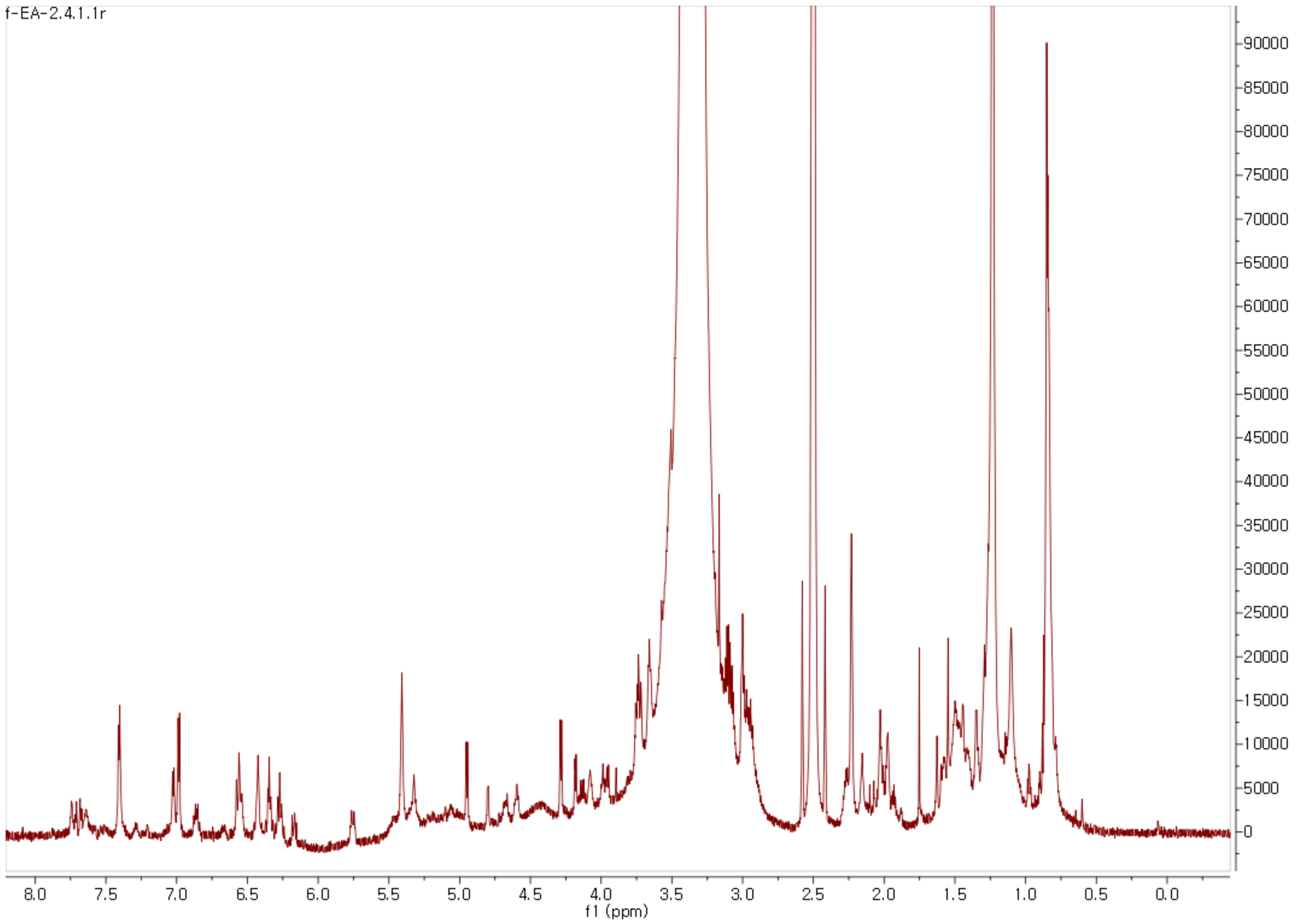

Figure S12. The ^{1}H NMR (850 MHz, DMSO-d_6) spectrum of **2**

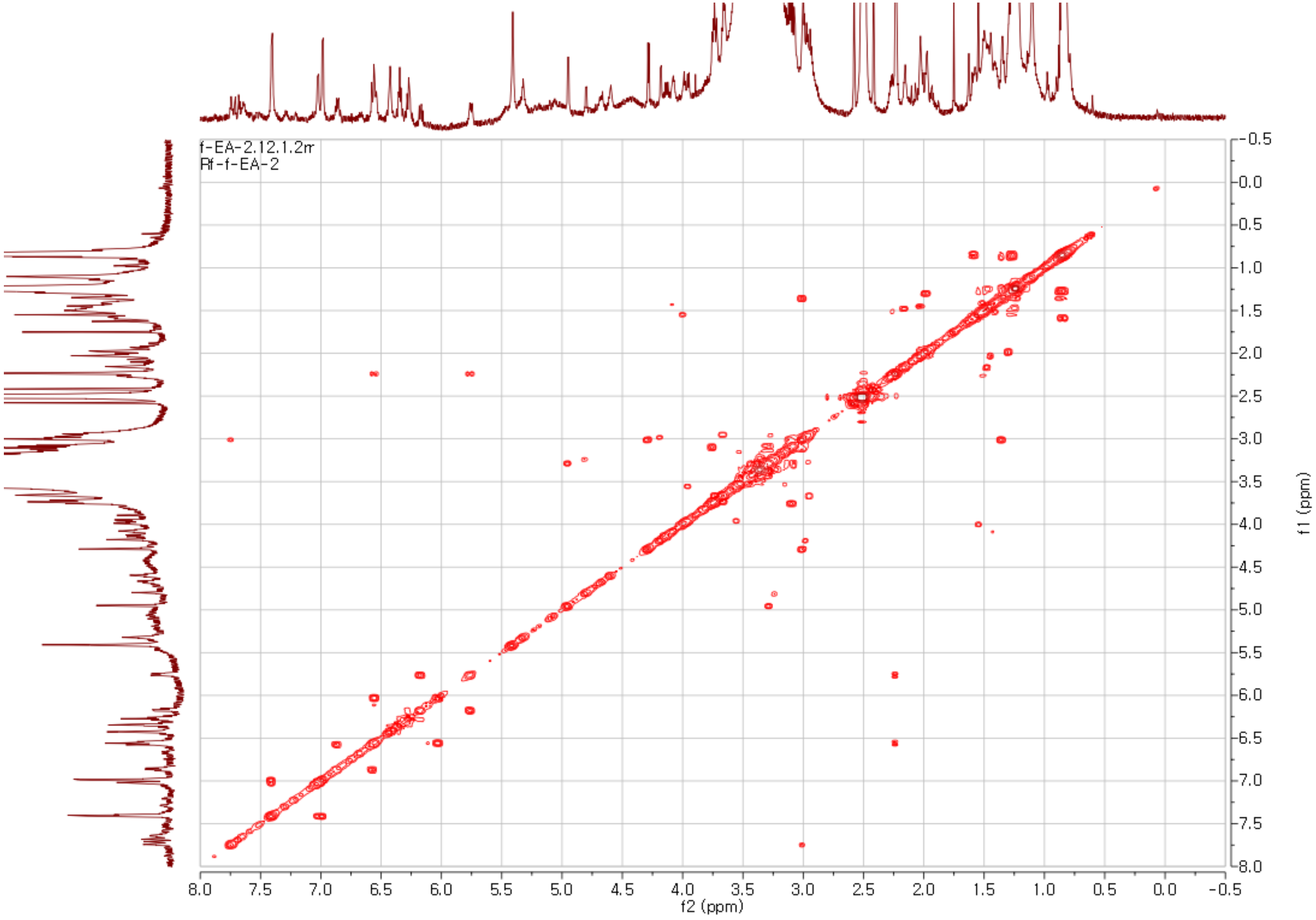

Figure S13. The ^{1}H-^{1}H COSY (850 MHz, DMSO-d_6) spectrum of **2**

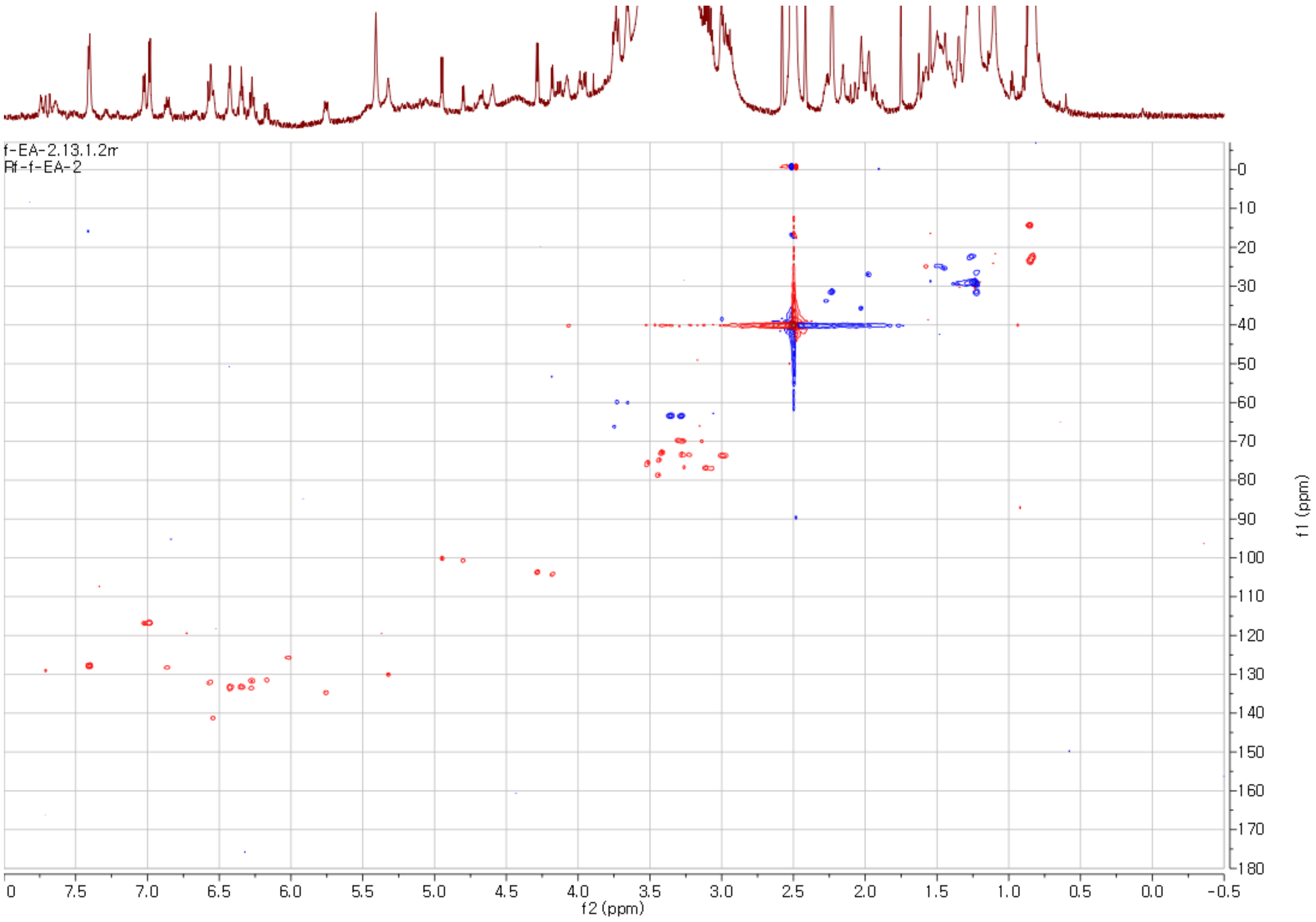

Figure S14. The HSQC (850 MHz, DMSO-d_6) spectrum of **2**

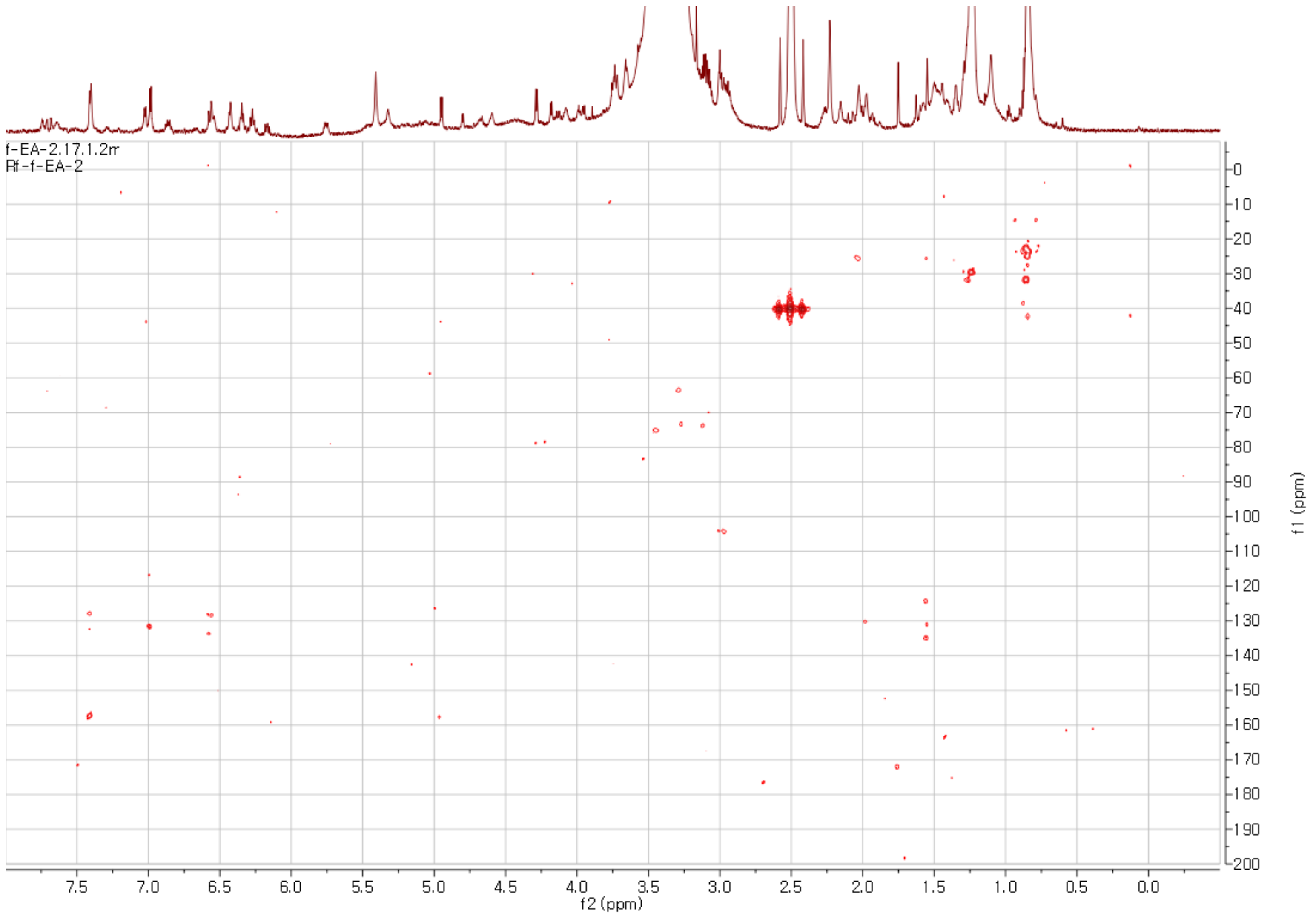

Figure S15. The HMBC (850 MHz, DMSO-d_6) spectrum of **2**

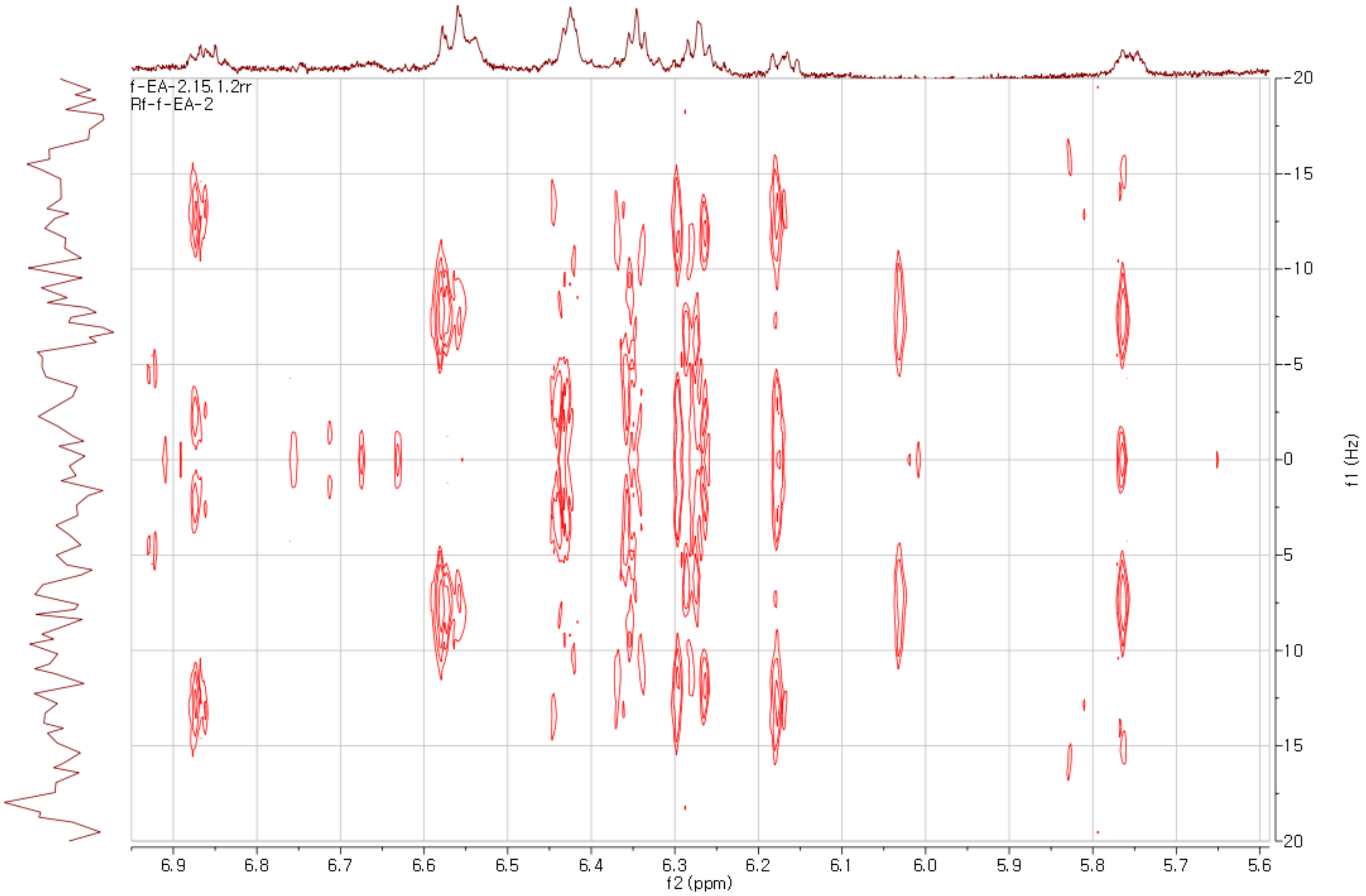

Figure S16. The *J*-resolved (850 MHz, DMSO-d_6) spectrum of **2**, expanded for the polyene region.

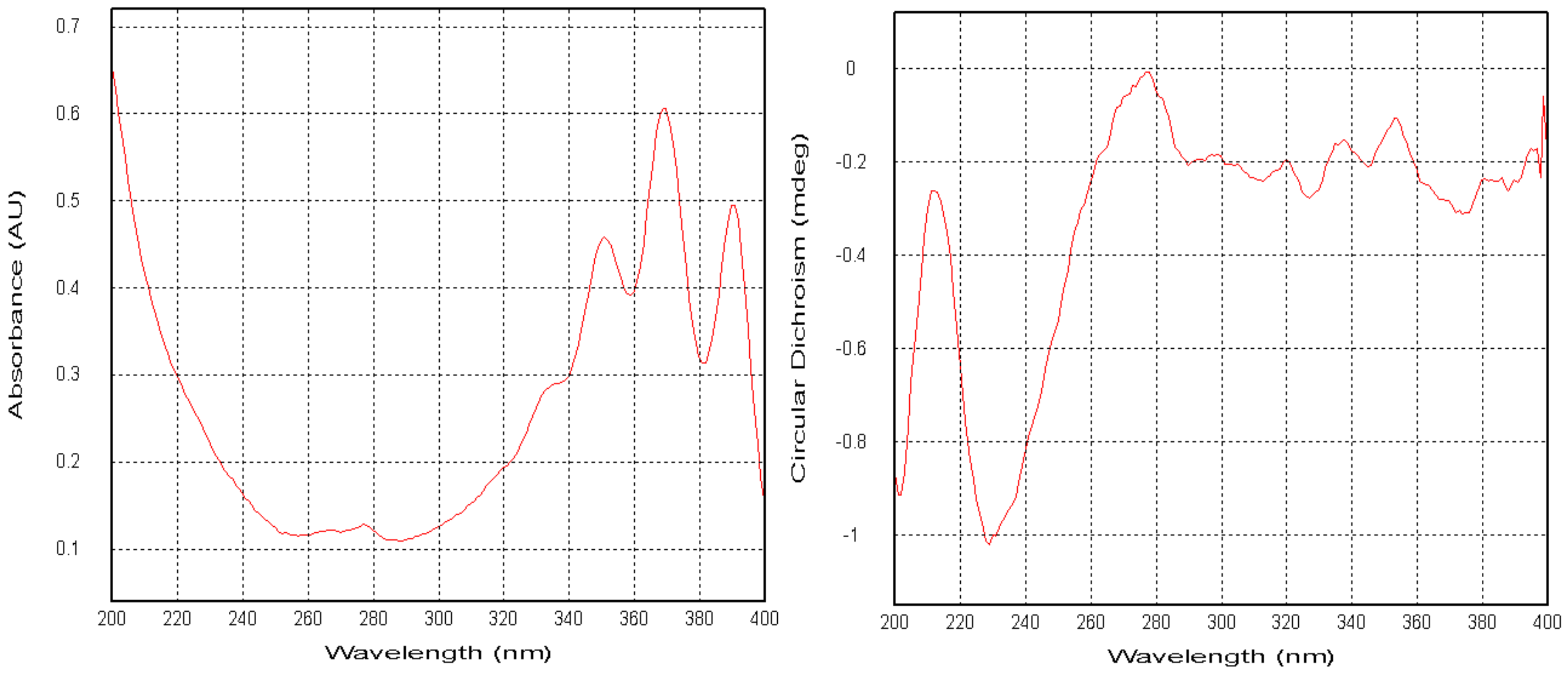

Figure S17. UV and CD spectra of **2**

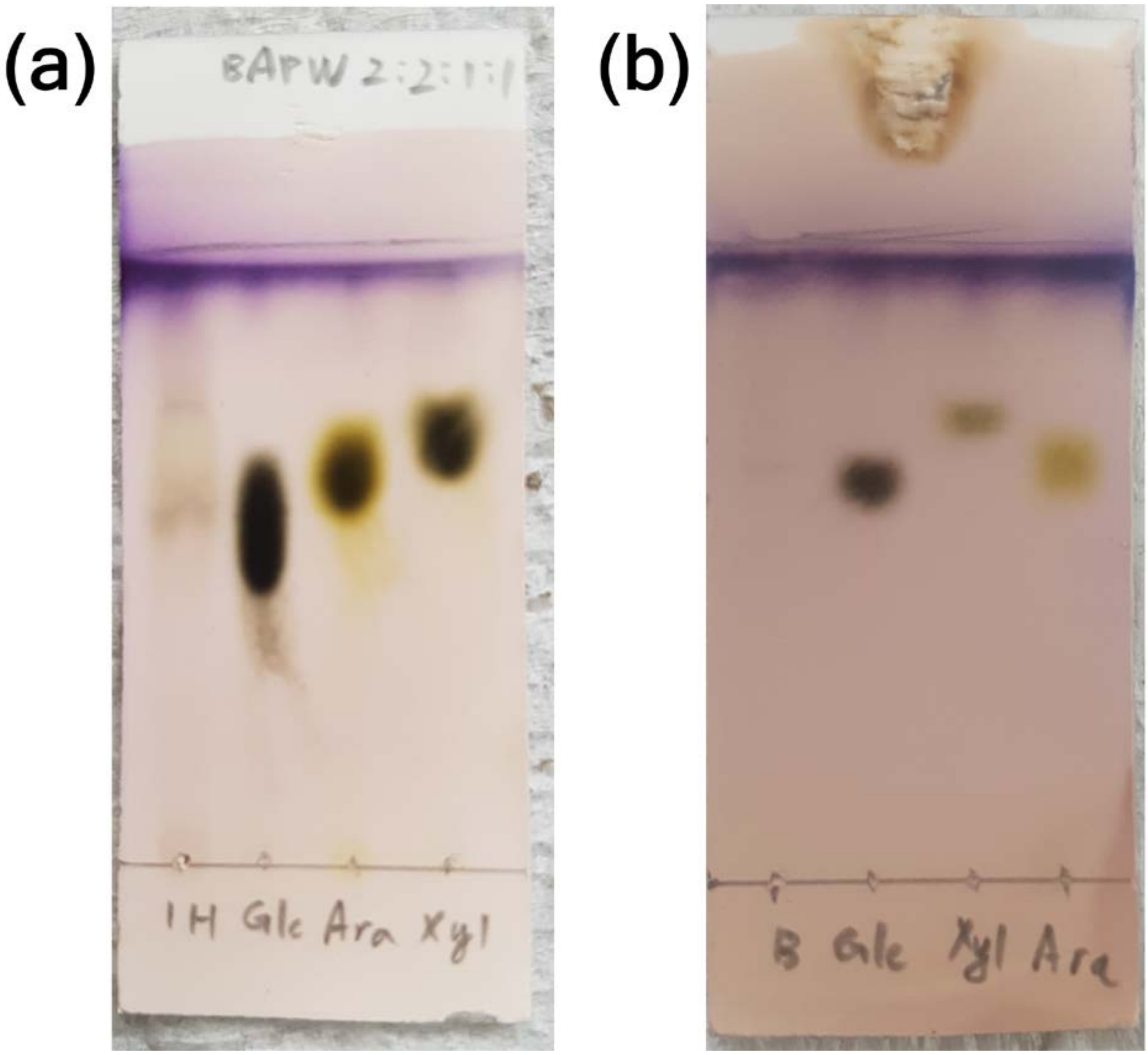

Figure S18. TLC for determining sugar moieties in hydrolysates of **1** (a) and **2** (b). Standards of glucose, arabinose, and xylose were spotted together.

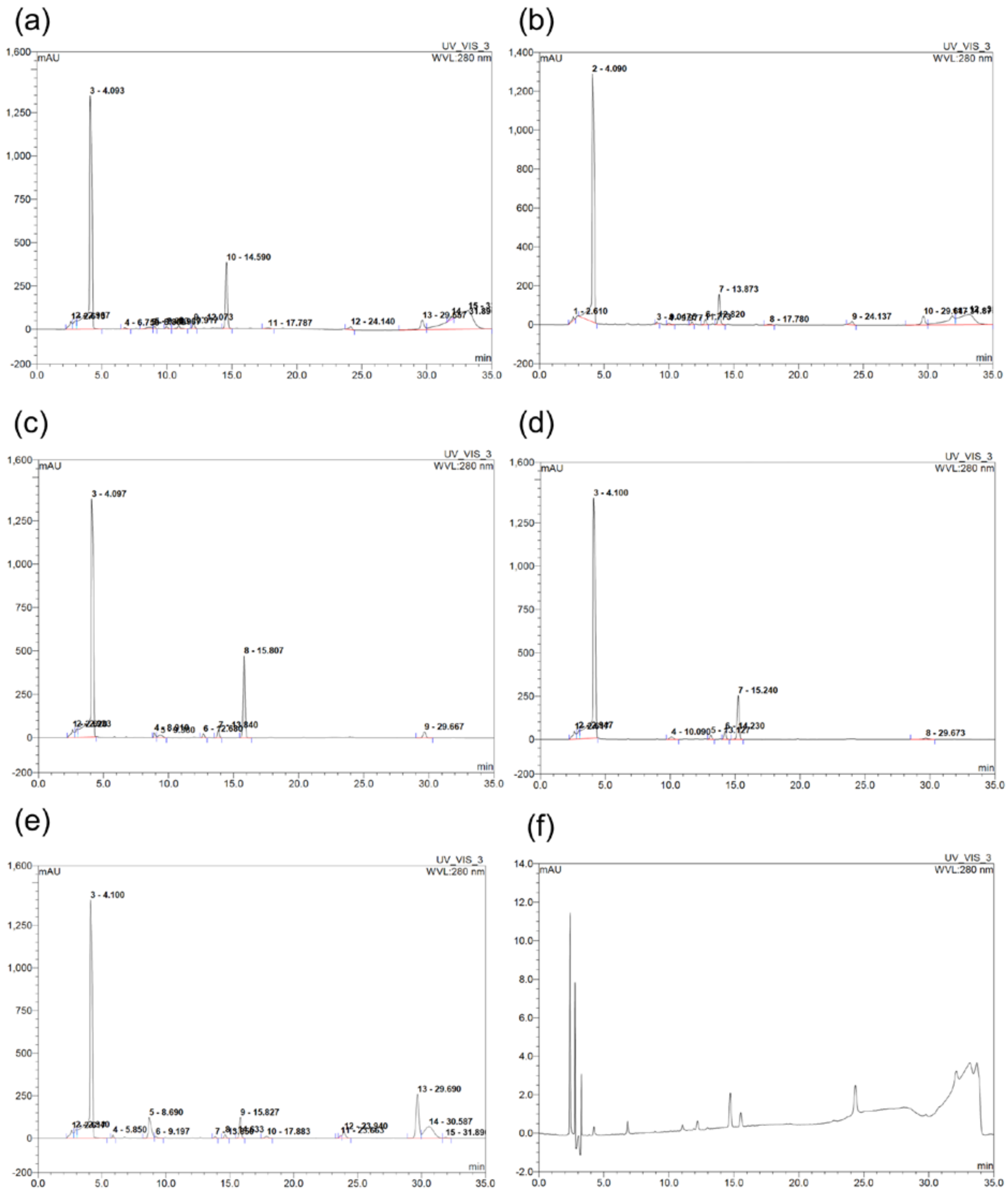

Figure S19. HPLC chromatograms for analyzing sugar derivatives in hydrolysates of **1** and **2**. (a) D-glucose standard derivative (b) (c) D-xylose standard derivative (d) L-xylose standard derivative (e) derivatized hydrolysate of compound **1** (f) derivatized hydrolysate of compound **2**.

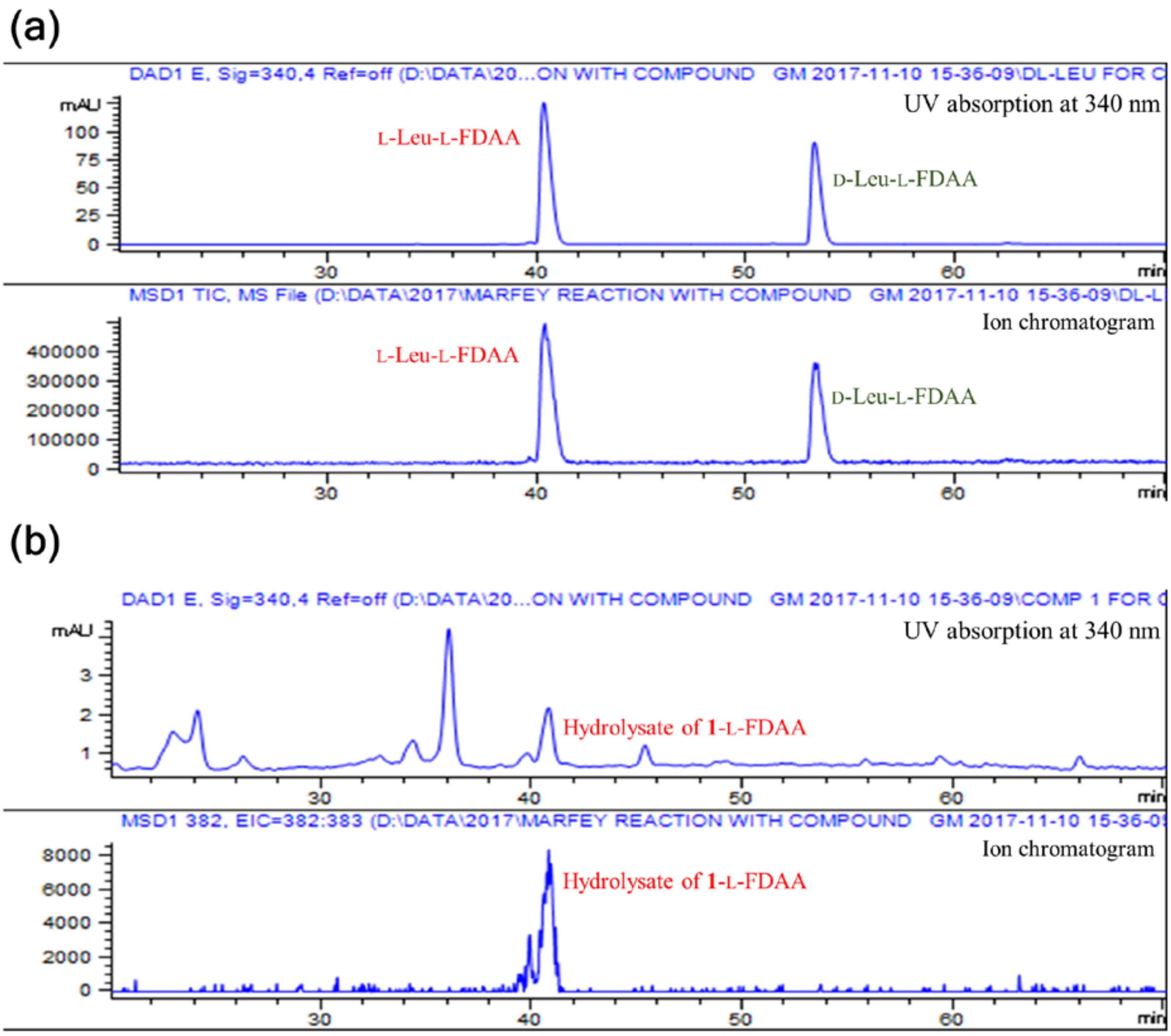

Figure S20. HPLC-DAD/MS chromatograms in C3 Marfey's analyses on the Leu unit of the hydrolysate of **1**. (a) L-FDAA derivatized L/D mixture of Leu, (b) L-FDAA derivatized hydrolysate of **1**.

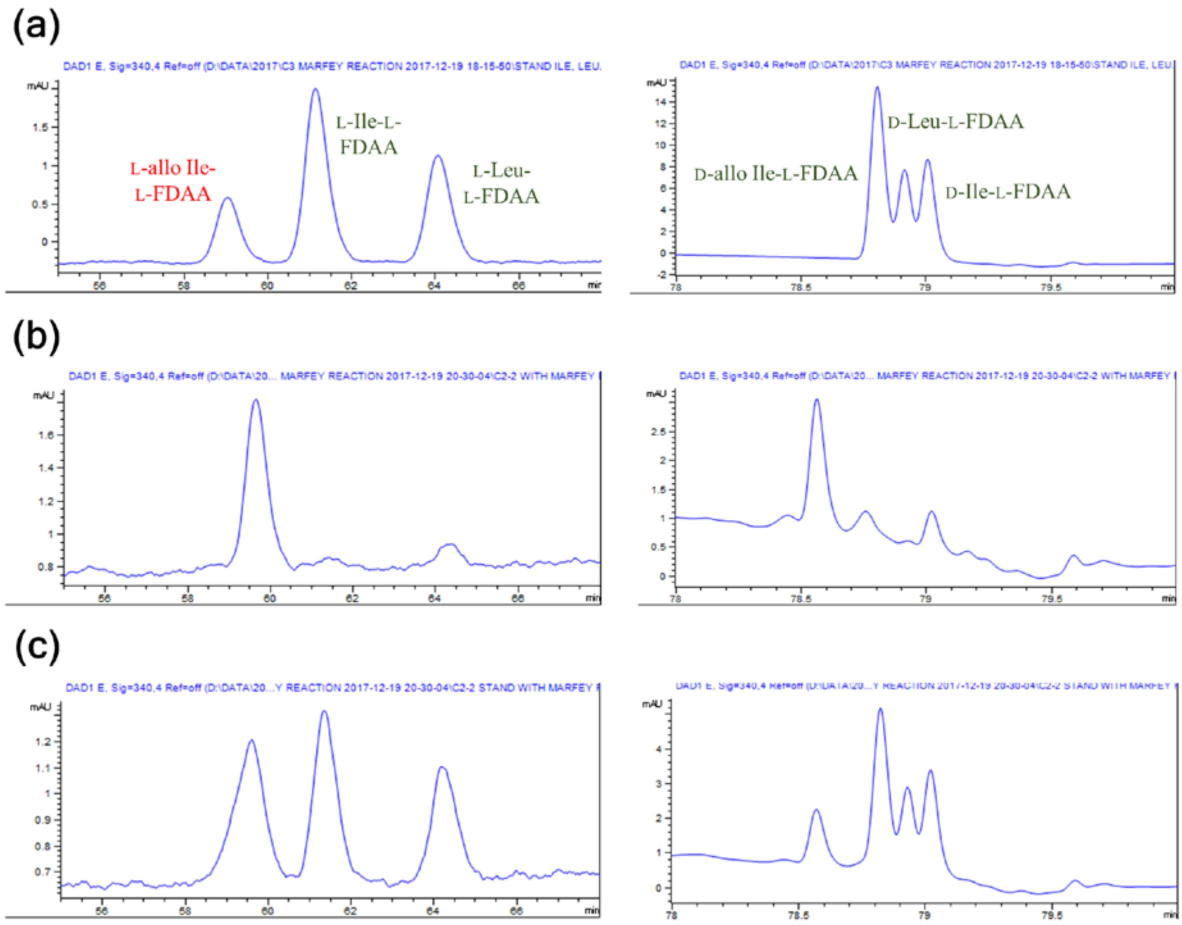

Figure S21. HPLC-UV (340 nm) chromatograms in C3 Marfey's analyses on the Leu and Ile units of the hydrolysate of **2**. (a) L-FDAA derivatized L/D standard mixtures of Leu/Ile/*allo*-Ile, (b) L-FDAA derivatized hydrolysate of **2**, (c) co-injected L-FDAA derivatized the standard mixture and hydrolysate of **2**.
